# Shotgun lipidomics of liver and brain tissue of Alzheimer’s disease model mice treated with acitretin

**DOI:** 10.1038/s41598-021-94706-3

**Published:** 2021-07-27

**Authors:** Anna A. Lauer, Daniel Janitschke, Malena dos Santos Guilherme, Vu Thu Thuy Nguyen, Cornel M. Bachmann, Sen Qiao, Bianca Schrul, Ulrich Boehm, Heike S. Grimm, Tobias Hartmann, Kristina Endres, Marcus O. W. Grimm

**Affiliations:** 1 Experimental Neurology, Saarland University, Homburg, Saar Germany; 2 Department of Psychiatry and Psychotherapy, University Medical Center Johannes Gutenberg-University, Mainz, Germany; 3 Experimental Pharmacology, Center for Molecular Signaling (PZMS), Saarland University School of Medicine, Homburg, Germany; 4 Medical Biochemistry and Molecular Biology, Center for Molecular Signaling (PZMS), Faculty of Medicine, Saarland University, Homburg, Saar Germany; 5 Deutsches Institut Für Demenzprävention (DIDP), Neurodegeneration and Neurobiology, Saarland University, Homburg, Saar Germany

**Keywords:** Alzheimer's disease, Lipidomics

## Abstract

Alzheimer’s disease (AD) is a very frequent neurodegenerative disorder characterized by an accumulation of amyloid-β (Aβ). Acitretin, a retinoid-derivative and approved treatment for Psoriasis vulgaris, increases non-amyloidogenic Amyloid-Precursor-Protein-(APP)-processing, prevents Aβ-production and elicits cognitive improvement in AD mouse models. As an unintended side effect, acitretin could result in hyperlipidemia. Here, we analyzed the impact of acitretin on the lipidome in brain and liver tissue in the 5xFAD mouse-model. In line with literature, triglycerides were increased in liver accompanied by increased PCaa, plasmalogens and acyl-carnitines, whereas SM-species were decreased. In brain, these effects were partially enhanced or similar but also inverted. While for SM and plasmalogens similar effects were found, PCaa, TAG and acyl-carnitines showed an inverse effect in both tissues. Our findings emphasize, that potential pharmaceuticals to treat AD should be carefully monitored with respect to lipid-homeostasis because APP-processing itself modulates lipid-metabolism and medication might result in further and unexpected changes. Moreover, deducing effects of brain lipid-homeostasis from results obtained for other tissues should be considered cautiously. With respect to acitretin, the increase in brain plasmalogens might display a further positive probability in AD-treatment, while other results, such as decreased SM, indicate the need of medical surveillance for treated patients.

## Introduction

Retinoids display various important functions in the body, such as regulating developmental processes, being central to vision, balancing proliferation as well as differentiation and response of the immune system (reviewed e.g. by^1^). Therefore, it is obvious that retinoid metabolites and synthetic molecules related to them are of pharmaceutical value. Vitamin A is well-known as a nutritional supplement important to be controlled during pregnancy^2^ and the use of retinoid-based drugs in inflammatory skin diseases such as psoriasis or ichthyosis has been established for decades (for a pharmacologic and clinical review on acitretin usage see^3^). One of the synthetic retinoids prescribed for severe psoriasis—also in the elderly population—is acitretin^4^. This aromatic derivative has also been identified as a potential drug for treating Alzheimer’s disease (AD) in a re-purposing screening attempt^5^. It showed in vitro but also in vivo favorable effects on hallmarks of the disease: for example, it enhanced α-secretase expression and activity in human neuronal cells and in mouse models of the disease^6,7^. This enzyme has been demonstrated to be protective against neurotoxic pathways of the disease such as the synthesis of amyloid-β (Aβ) peptides and e.g. prevents plaque formation in AD model mice^8^. Acitretin treatment also resulted in amelioration of behavioral deficits of these mice^7^. These observations led to the idea of using retinoids as therapeutic drugs in human AD patients^9,10^. In a first small study, the efficacy of α-secretase enhancement in patients with mild to moderate disease could be shown by an increase of the specific cleavage product from the amyloid precursor protein (APP) in CSF after 30 days treatment with a dosage of 30 mg/day^11^. However, despite all these supporting facts, implementation of acitretin into clinical daily usage is complicated because of potential side effects—especially in the elderly target population that might suffer from comorbidities and poly-medication. The most common side effects observed from patients treated with acitretin for skin disease are elevation in serum triglycerides and to a lesser extent increased levels of cholesterol and liver transaminase^12,13^. Psoriasis patients subjected to a fixed tapering dose for four weeks showed for instance an increase of triglycerides and VLDL with a maximum change in the first and second week of treatment, while LDL and cholesterol only changed maximally in the second half of the treatment period^14^. Early attempts in the 1990s tried to decipher the underlying mechanisms: Using Ng57Bln hairless mice, an acitretin dosage-dependent change in hepatic lyso-phosphatidylcholine (lyso-PC) could be shown by two-dimensional thin layer chromatography (TLC). 3 mg/kg acitretin per day lead to a decrease, while 10 mg/kg per day lead to an increase^15^. Phosphatidylcholine (PC) showed the opposite direction of effect. Similar results were obtained when acitretin was administered to rats orally for six weeks^16^. A reduction in hepatic microsomal protein content and aminopyrine-N-demethylase, an increase in P450, elevated ethoxyresorufin-O-deethylase and the in vitro potential of its binding to LDL and HDL^17^, all together indicate an interference of acitretin with liver metabolism. Therefore, an explicit warning is given in instruction leaflets and prescription advices for acitretin. However, the effect on liver lipid homeostasis has not been systematically investigated to clarify its exact impact. Likewise, a potential impact on brain lipid metabolism has not been investigated yet. As hyperlipidemia and altered brain lipid levels are discussed as risk factors for AD (e.g.^18,19^), such analyses are highly demanded with regard to acitretin’s potential usage in AD treatment. Interestingly, it has been reported that AD also induces changes in lipid homeostasis itself and that APP processing products are important regulators of lipid metabolism (reviewed e.g. in^20,21^). Acitretin might therefore interfere with, or aggravate, or attenuate these AD-dependent changes in lipid homeostasis. Here, we analyzed the influence of acitretin administration on lipid composition of liver and brain in mid-age AD model mice by semi-quantitative shotgun mass spectrometry to clarify these probable impacts. In this shot gun mass spectrometry approach we focused on sphingomyeline, carnitine, phosphatidylcholine-plasmalogens, phosphatidylcholine, lyso-phosphatidylcholine, and triacylglycerides, because these lipids are known to be affected by AD or APP processing or altered in serum samples of patients treated with acitretin^21–28^. Moreover, we focused on lipids, which were not influenced by matrix effects in our approach, having a low intra- and inter-day variance and a high yield in the utilized extraction method.

## Results

### Lipid analysis in brain and liver tissue of acitretin-treated 5xFAD transgenic mice

**Figure 1:**
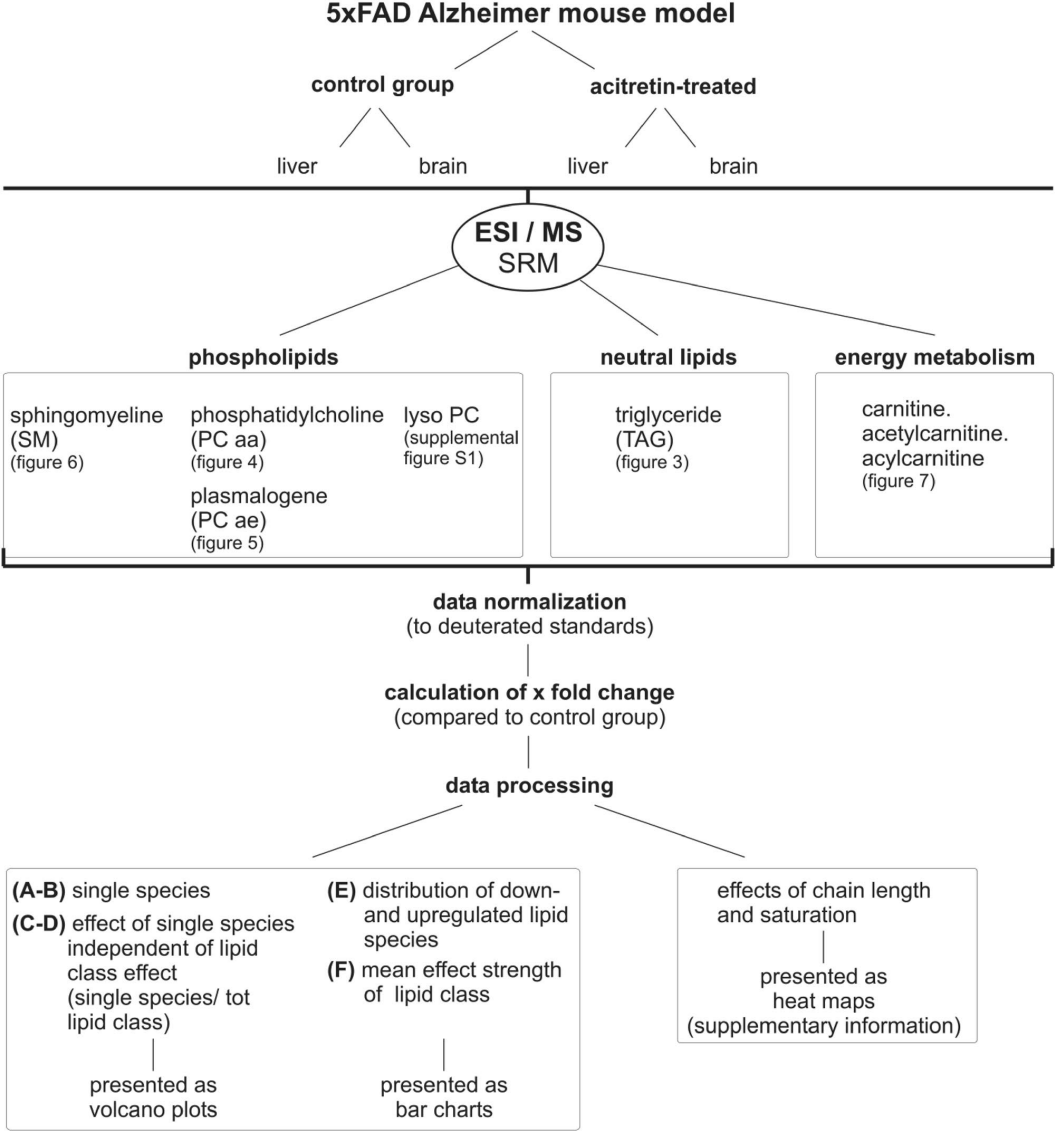
Schematic overview of the study design. FAD: Familial Alzheimer´s Disease. ESI/MS SRM: Electrospray Ionisation/Mass Spectrometry Selected Reaction Monitoring. Tot: total.

To investigate the possible impact of acitretin on lipid homeostasis with respect to AD, we treated 30 week old 5xFAD mice with acitretin under experimental conditions, which have been previously shown to ameliorate behavioral or cognitive deficits in these AD model mice^7^. In total, 750 parameters were semi-quantitatively measured by shotgun mass spectrometry (MS) in brain and liver tissue of acitretin-treated transgenic mice and compared to transgenic animals not treated with acitretin (control group) (Fig. 1). The analyzed parameters include phospholipids (sphingomyeline (SM), phosphatidylcholine (PCaa), plasmalogens (PCae), lyso-PC), neutral lipids (triglycerides (TAG)) as well as carnitine, acyl-carnitine and acetyl-carnitine, which are involved in the carnitine carrier system, that is responsible for fatty acid (FA) transport from the cytosol to the mitochondria, where the FAs esterified to carnitines are metabolized by β-oxidation (see supplemental Figure S6).

The obtained data were normalized to deuterated standards and calculated as x-fold change compared to the control group. As described in the material and method section at least one deuterated standard of each lipid class was added to the sample before lipid extraction to minimize variability caused by different extraction efficiency. Lipid extraction was checked and comparable results such as reported before were obtained (average recovery rate = 80.7%, see supplemental Figure S5C)^24^. The intra- and inter-day variance was constantly measured with an average of 6.5% (see supplemental Figure S5A). Shotgun lipidomics is in principle susceptible for matrix effects. In order to estimate the impact of these potential matrix effects on the obtained results, the ratio between the deuterated lipid standards in presence of lipid extracts from acitretin-treated mice or the control group, respectively, were measured. The change in the ratio provided a maximum of 3.2% and an average of 1.4% (shown in supplemental Figure S5B). For triacylglycerides (TAGs) the sum of the FAs bound to the sn1, sn2 and sn3 position, for PCaa and PCae the FAs bound to the sn1 and sn2 position are presented. For SM, SM with 18:1 in the sn1 position was measured.

**Figure 2:**
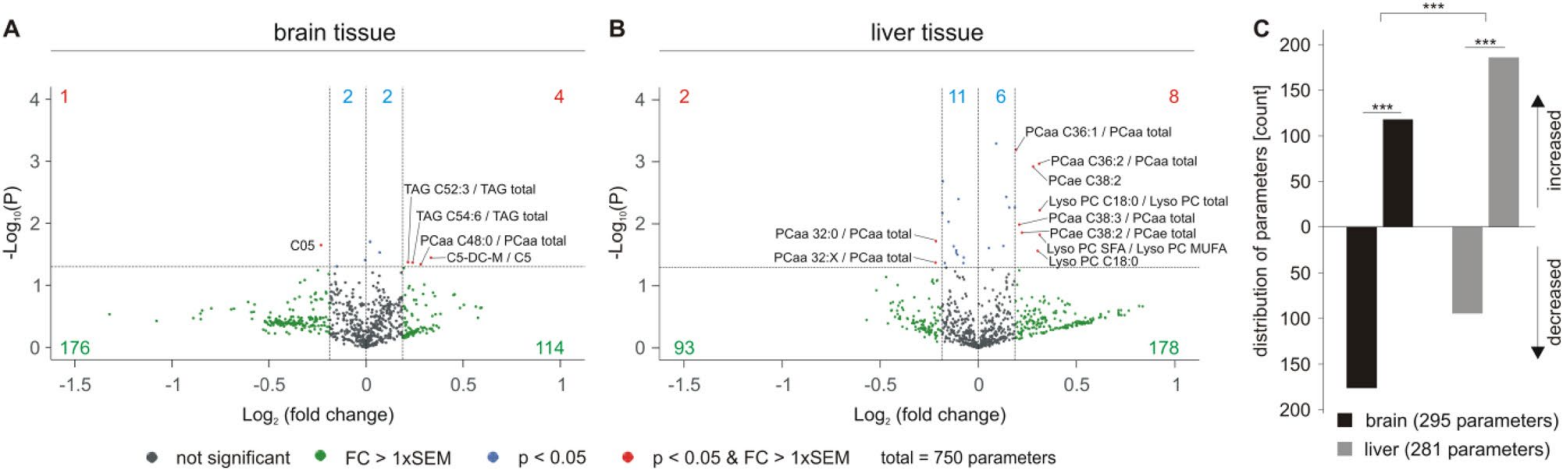
Lipid changes in acitretin-treated AD model mice compared to AD transgenic mice not treated with acitretin. Fold changes of 750 parameters are plotted logarithmically against the *p* value (−Log_10_) for brain (**A**) and liver tissue (**B**) of 5xFAD mice. Those lipid species without significant changes are represented as grey dots, those with a fold change greater than the average SEM as green dots. Species with a significant (*p* < 0.05) fold change are highlighted in blue and those with a *p* value < 0.05 and a fold change greater than the SEM are marked as red dots in the volcano plot. The exact number of changed parameters in every part of the volcano plot is written in the appropriate color. (**C**) Distribution of parameters with a fold change greater than the average SEM represented as number of de- and increased parameters in brain and liver tissue in a bar chart. The total number of parameters used in the statistical analysis is indicated. Statistical significance of the observed shifts in de- and increased parameters in brain or liver tissue was calculated using binomial test. For analysis of significant differences in lipid distribution between brain and liver tissue Fisher´s exact test was used.

The volcano plots shown in Fig. 2 represent lipid changes in acitretin-treated AD model mice compared to control-treated mice. Throughout the complete manuscript, the volcano plots are divided in eight squares and analyzed accordingly. Lipid parameters that showed an increase or decrease after acitretin treatment, which was smaller than the average standard error of the mean (SEM) and not significant, were marked as grey dots and were not further analyzed. Non-significant changes with a magnitude of effect greater than the average SEM were marked as green dots. Taking into consideration that the definition of significance has an arbitrary aspect and depends on the statistical method (e.g. which kind of type one error correction) and the number of samples, we decided also to include lipid parameters having an effect stronger than the mean SEM of the corresponding lipid class but not reaching significance. In this context, we would like to point out that beside the significance of a single lipid species the distribution of lipids with chemical similarities should be recognized and might be relevant. For example, if 80% of a single lipid species out of one lipid group show an alteration in the same direction without reaching significance for the single species, this result should also be recognized. By just plotting or focusing on the significant parameters, this information would be lost; such changes, however, have to be verified in further studies. Red dots represent the significantly changed parameters with a magnitude of effect more than the average SEM and blue dots are significantly changed but have an effect strength less than the average SEM. Similar to the parameters, which are not significant but with a high effect strength, also these parameters might be relevant but are especially vulnerable to over-interpretation and therefore need to be verified in further studies as well. The potential caveats are emphasized further in the discussion.

The analysis of 750 parameters by mass spectrometry in brain tissue of acitretin-treated 5xFAD mice compared to the control group revealed that 114 lipid parameters tended to increase whereas 176 parameters tended to decrease with an effect strength greater than the SEM (Fig. 2A). Six parameters were significantly increased (four out of the SEM and two within the SEM) and three parameters significantly decreased (one out of the SEM and two within the SEM). In liver tissue, 178 parameters tended to increase, whereas only 93 parameters showed a trend to be decreased upon acitretin treatment (Fig. 2B). In total, 14 parameters were significantly increased and 13 parameters significantly decreased. Notably, the observed shifts in down- and upregulated parameters were highly significant for both, brain and liver tissue (*p* ≤ 0.001) (Fig. 2C), and highly significant changes exist by comparing brain with liver tissue utilizing the Fisher´s exact test. Upon acitretin treatment, more lipid parameters increased in liver tissue than in brain tissue, but conversely more parameters decreased in brain tissue than in liver tissue. One might conclude that in accordance with the function of the liver, for some lipids the liver might provide lacking or decreased lipid species in brain to compensate the observed changes in the brain.

### Mass spectrometry analysis of triacylglycerides in brain and liver tissue of acitretin-treated AD transgenic mice

As mentioned above, to identify lipid classes and single lipid species that are affected by acitretin treatment, we analyzed triacylglycerides (TAG), phosphatidylcholine (PCaa), plasmalogens (PCae), lyso-PC, sphingomyelin (SM), and carnitines. In principle, observed alterations of a single lipid can be due to the head group of a lipid, determining the lipid class, or due to the FA bound to the backbone of the lipid. To elucidate if the FAs attenuate or intensify the effect observed for the lipid class we decided to additionally normalize each lipid species to the average lipid class effect.

**Figure 3:**
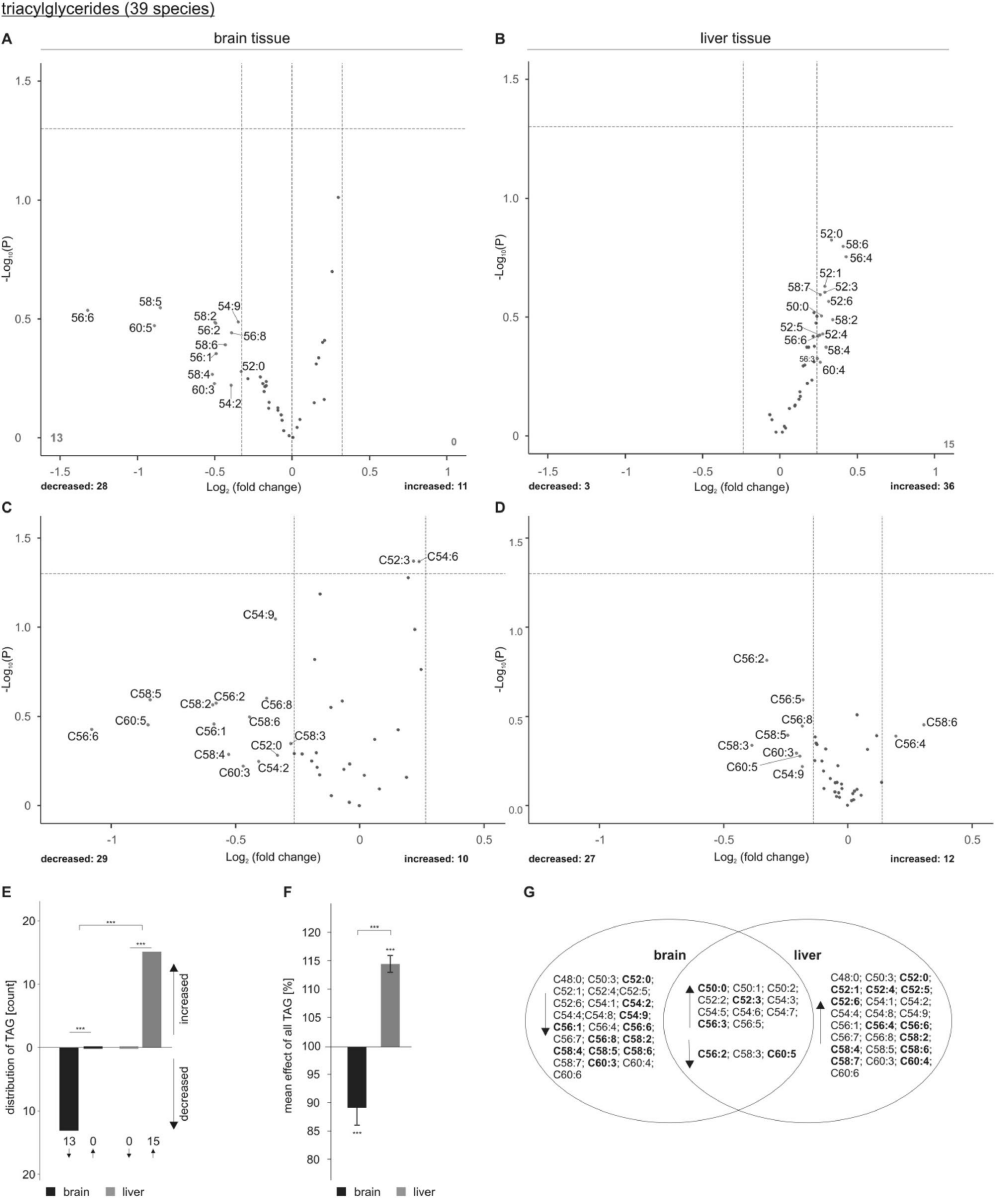

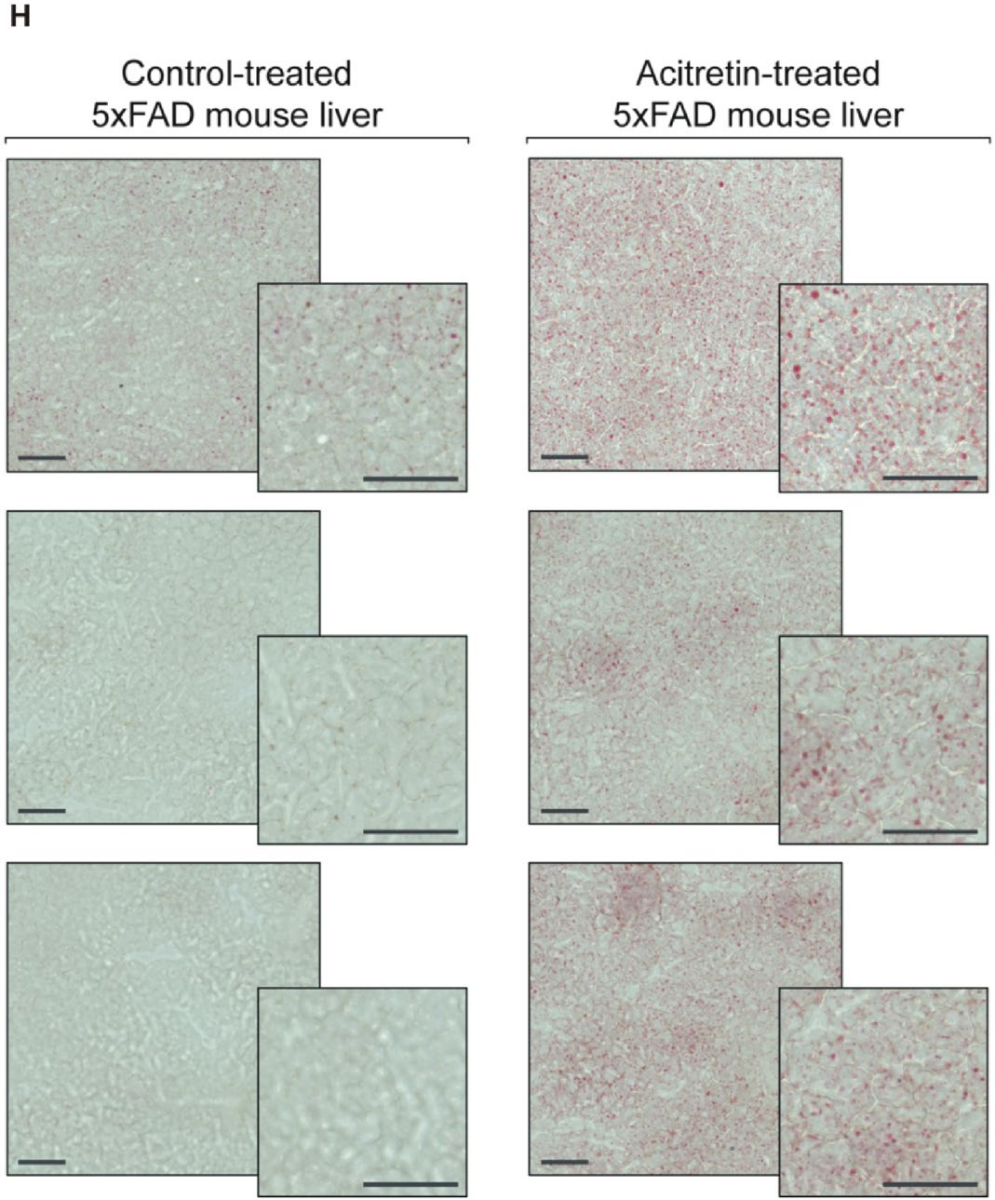
Changed triacylglyceride (TAG) levels in 5xFAD brain and liver tissue after acitretin treatment. Fold changes of single TAG species in brain and liver tissue are shown as volcano plots on the top (**A**: brain tissue, **B**: liver tissue) and the effects of single species independent of lipid class effect for the analyzed species are presented as appropriate volcano plot on the bottom (**C**: brain tissue, **D**: liver tissue). Structure and labeling of the volcano plots are according to Fig. 2. (**E**) Distribution of TAG species represented as number of de- and increased parameters in brain and liver tissue in a bar chart. Statistical significance of the observed shifts in de- and increased parameters in brain or liver tissue was calculated using binomial test. For analysis of significant differences in TAG distribution between brain and liver tissue Fisher´s exact test was used. (**F**) Mean effects on all TAGs in brain and liver tissue are shown in a bar chart. Statistical significance of the mean effects in brain and liver tissue compared to control mice was calculated using one-sample t-test and differences in the mean effects between the two analyzed tissues were calculated using two sample t-test. (**G**) Venn diagram showing exclusively changed as well as overlapping TAG species in brain and liver tissue. Species with a fold change greater than the SEM are highlighted in bold. (**H**) Oil Red O staining of lipid droplets in liver sections from control-treated (left) and acitretin-treated (right) mice. Maximum intensity projections of 1 µm z-sections of six independent samples from six animals (three control-treated and three acitretin-treated mice) are shown. Scale bar: 50 µm.

The volcano plots in Fig. 3A and B illustrate the analysis of 39 TAG species in brain and liver tissue of acitretin-treated transgenic animals normalized to the control group, whereas the volcano plots shown in Fig. 3C and D show changes in specific lipid species independent of the lipid class group effect. Interestingly, we observed that 28 TAG species were decreased and 11 TAG species increased in brain tissue. 13 out of the 28 decreased TAG species revealed an effect strength higher than the average SEM, whereas all increased TAG species showed an effect strength within the SEM. An inverse effect was observed for liver tissue: 36 TAG species were increased (15 out of the average SEM), whereas only three TAG species were decreased (all within the SEM). The observed alterations in the number of de- or increased TAG species (effect strength higher than the average SEM) were highly significant for brain and liver, respectively. Moreover, the differences between liver and brain tissues are also highly significant (Fig. 3E). The distinct effects of acitretin on TAGs in brain and liver is further substantiated by the finding that the mean effect of all analyzed TAG species was significantly reduced in brain and significantly increased in liver (Fig. 3F). The Venn diagram shows overlapping species and those who are de- or increased in brain or liver tissue exclusively (Fig. 3G). The acitretin-induced elevation in TAGs in liver tissue is in line with the reported prevalence of nonalcoholic fatty liver disease in patients treated with acitretin and the findings that acitretin is described to cause hyperlipidemia^14,29,30^. The effect of acitretin treatment on the total amount of the analyzed TAG species is shown in supplementary Figure S7.

Normalization to the total TAG content revealed that TAG containing polyunsaturated fatty acids (PUFAs) are mostly affected by acitretin treatment in brain tissue. We found reduced level of e.g. TAG C54:9, C58:5, C60:5, C56:6, C58:4, C58:6, C56:8 and C56:6. These TAG might include, in accordance to literature, the omega-3 PUFAs docosahexaenoic acid (C22:6) and eicosapentaenoic acid (C20:5), which are discussed to play a role in AD pathology^22,31–34^. Although total TAG were increased in liver tissue upon acitretin treatment, we also found individual TAG species to be decreased. Similar to brain tissue e.g. TAG C56:8, C58:5, C60:5 and C54:9 were decreased after normalization to total TAG content, indicating that it might be beneficial to supplement DHA and EPA to patients treated with acitretin to attenuate these effects.

In cells, the majority of TAG is stored in lipid droplets. To test whether acitretin treatment leads to enhanced TAG storage in lipid droplets, we performed Oil Red O staining on liver sections. In line with the acitretin-dependent increase of TAGs, more Oil Red O staining and an increase in lipid droplets was detected upon acitretin treatment (see Fig. 3H).

## Alterations in phosphatidylcholine in acitretin-treated AD transgenic mice

**Figure 4:**
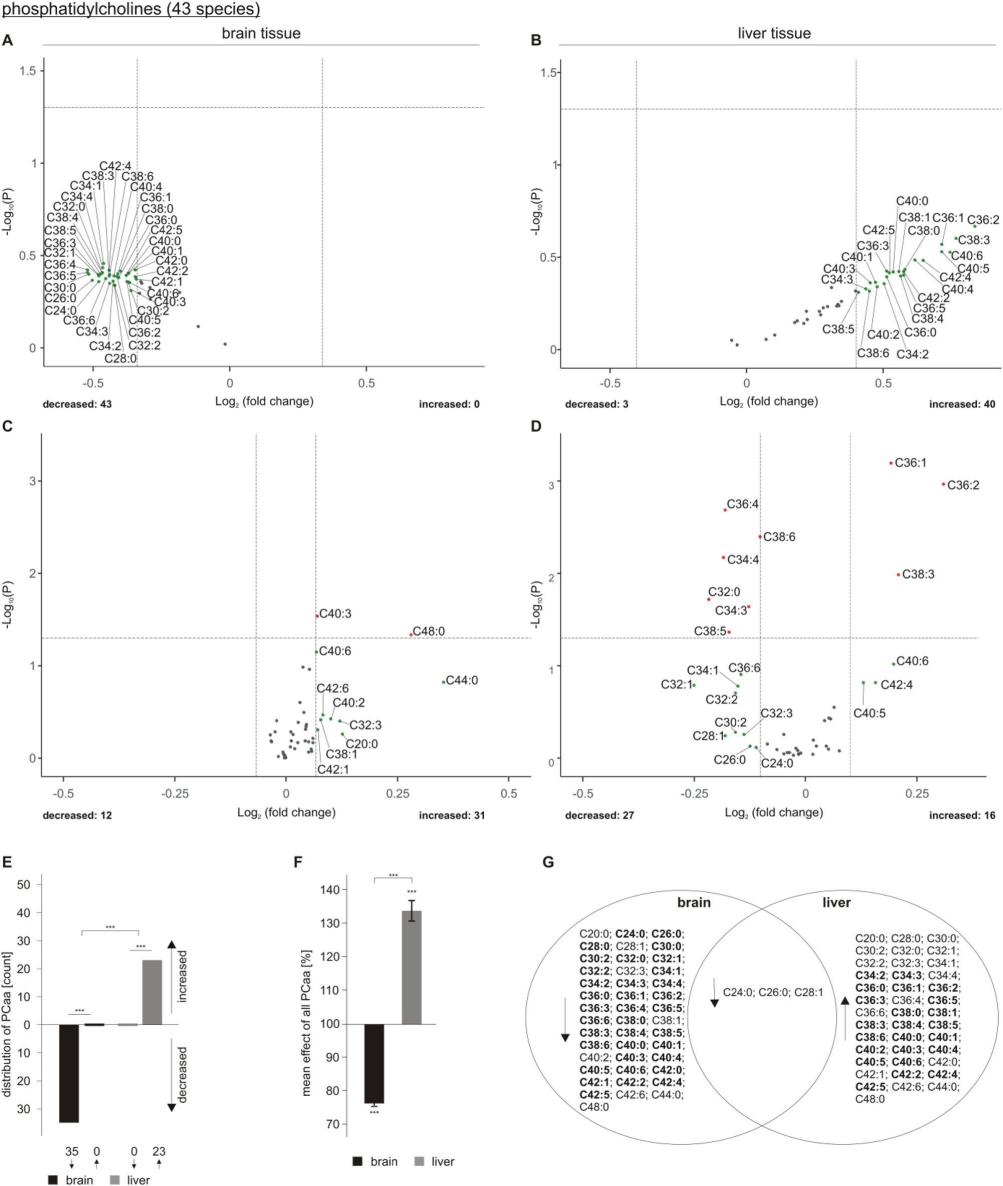
Changed phosphatidylcholine (PCaa) levels in 5xFAD brain and liver tissue after acitretin treatment. Fold changes of single PCaa species in brain and liver tissue are shown as volcano plots on the top (**A**: brain tissue, **B**: liver tissue) and the effects of single species independent of lipid class effect for the analyzed species are presented as appropriate volcano plot on the bottom (**C**: brain tissue, **D**: liver tissue). Structure and labeling of the volcano plots are according to Fig. 2. (**E**) Distribution of PCaa species represented as number of de- and increased parameters in brain and liver tissue in a bar chart. (**F**) Mean effects on all PCaa in brain and liver tissue are shown in a bar chart. Statistical significance for (**E**) and (**F**) was calculated according to Fig. 3. (**G**) Venn diagram showing exclusively changed as well as overlapping PCaa species in brain and liver tissue. Species with a fold change greater than the SEM are highlighted in bold.

TAGs are mainly stored in specialized cellular organelles, called lipid droplets, which are surrounded by monolayers consisting of phospholipids, e.g. phosphatidylcholine. Therefore, in line with previously published papers, revealing that TAG level correlates with lipid droplet formation and that PC synthesis correlates with lipid droplet expansion, one can assume that alterations in TAG might also have an impact on total PCaa level^35,36^. In line with the observed increase in total TAG content in liver tissue of acitretin-treated transgenic mice, we found 40 out of 43 analyzed PCaa species to be elevated, 23 out of them with an effect strength greater that the average SEM (Fig. 4B) whereas only three PCaa species tended to decrease. The effect of acitretin treatment on the total amount of the analyzed PCaa species is shown in supplementary Figure S7.

Normalized to total PCaa level, PCaa C36:1, C36:2 and C38:3 were significantly increased in liver tissue, whereas mainly PUFA-containing PCaa species were significantly decreased (PCaa C36:4, C38:6, C34:4, C34:3, C38:5) (Fig. 4D). Interestingly, PCaa 40:5 and PCaa 42:4 showed an increase higher than the average SEM, normalized (Fig. 4D) or not normalized to total PCaa content (Fig. 4B). As these PCaa species are reported to include the omega-6 PUFA arachidonic acid C20:4, this observation might be important with respect to acitretin-induced inflammatory processes in liver tissue.

All 43 analyzed PCaa species tended to decrease in brain tissue of AD model mice treated with acitretin (Fig. 4A); 40 out of them showed a strong trend out of the SEM. Normalized to the total PCaa content, we found no specific PCaa species to be significantly reduced in brain tissue of acitretin-treated 5xFAD mice (Fig. 4C), indicating that acitretin treatment rather leads to a reduction in total PCaa level in brain than affecting individual PCaa species.

In line with the observed alterations in the number of de- or increased TAG species in brain and liver tissue upon acitretin treatment, also the changes in the number of PCaa species that are decreased in brain and upregulated in liver, respectively, were highly significant (Fig. 4E). Likewise, the obtained differences between liver and brain were highly significant. The opposite effect of acitretin on PCaa species in brain and liver tissue was also found by analyzing the mean amount of all PCaa species that was significantly decreased in brain and significantly elevated in liver (Fig. 4F). The Venn diagram shows overlapping species and those who are de- or increased in brain or liver tissue exclusively (Fig. 4G).

## Effect of acitretin on plasmalogens in AD transgenic mice

**Figure 5:**
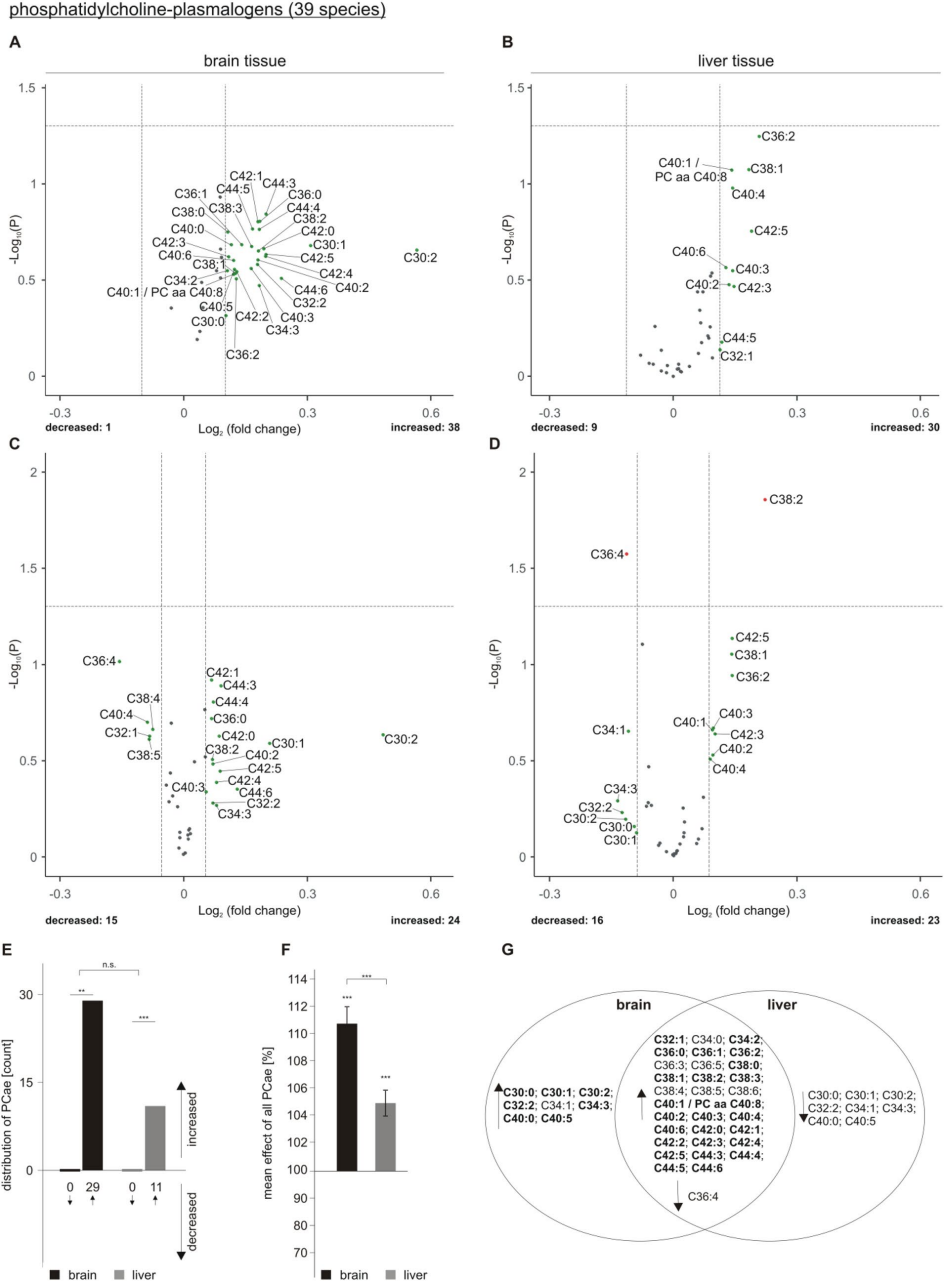
Changed phosphatidylcholine-plasmalogen (PCae) levels in 5xFAD brain and liver tissue after acitretin treatment. Fold changes of single PCae species in brain and liver tissue are shown as volcano plots at the top (**A**: brain tissue, **B**: liver tissue) and the effects of single species independent of lipid class effect for the analyzed species are presented as appropriate volcano plot at the bottom (**C**: brain tissue, **D**: liver tissue). Structure and labeling of the volcano plots are according to Fig. 2. (**E**) Distribution of PCae species represented as number of de- and increased parameters in brain and liver tissue in a bar chart. (**F**) Mean effects on all PCae in brain and liver tissue are shown in a bar chart. Statistical significance for (**E**) and (**F**) was calculated according to Fig. 3. (**G**) Venn diagram showing exclusively changed as well as overlapping PCae species in brain and liver tissue. Species with a fold change greater than the SEM are highlighted in bold.

In contrast to PCaa, we found that plasmalogens (PCae) were increased in brain tissue as a result of acitretin treatment. 38 out of the 39 analyzed PCae species tended to be elevated, 29 with an effect strength higher than the average SEM (Fig. 5A). An increase in PCae could be also detected for liver tissue: 30 PCae species were elevated (eleven out of them with an effect strength higher than the average SEM), whereas nine PCae species were decreased (all within the average SEM) (Fig. 5B). Normalized to total PCae level, PCae C38:2 was found to be significantly increased in liver tissue of acitretin-treated transgenic animals and PCae C36:4 significantly decreased (Fig. 5D). These PCae species also showed a strong trend to be altered in brain tissue: PCae C38:2 showed an increase with an effect strength higher than the average SEM and PCae C36:4 showed a strong trend to be decreased in brain (Fig. 5C), but also did not reach significance. The effect of acitretin treatment on the total amount of the analyzed PCae species is shown in supplementary Figure S7.

The shift of PCae species to be upregulated upon acitretin treatment was highly significant for both tissues, brain and liver (Fig. 5E). No significant effect in the number of upregulated PCae species exists between liver and brain. The mean effect of all analyzed PCae species revealed a significant increase to 110.86% ± 1.25% in brain and a significant elevation to 105.01% ± 0.95% in liver upon acitretin treatment in transgenic animals (Fig. 5F). The observed effect of acitretin on the mean of all PCae species was found to be significantly stronger in brain than in liver. The Venn diagram shows overlapping species and those who are de- or increased in brain or liver tissue exclusively (Fig. 5G).

Taken into consideration that plasmalogens were reported to be decreased in AD^24,37–39^, this observed increase in plasmalogens might be another positive aspect in a potential therapeutical use of acitretin with respect to AD. However, further studies are needed to clarify this aspect in more detail and in particular in human patients.

### Mass spectrometry analysis of lyso-phosphatidylcholine in brain and liver tissue of acitretin-treated AD transgenic mice

Analyzing lyso-PC species in brain and liver tissue of acitretin-treated AD transgenic mice revealed a slight decrease in lyso-PC species for both tissues. In brain, 13 out of 22 analyzed lyso-PC species tended to decrease whereas nine lyso-PC species showed a trend to increase, however the observed effect was not statistical significant (supplement Figure S1E). A slight but not significant effect was also found by analyzing the mean effect of acitretin on all lyso-PC species (supplement Figure S1F). The four elevated lyso-PC species that showed an effect strength out of the average SEM were mainly lyso-PC species containing long chain FAs, C26:1, C28:0, C22:0 and C28:1 (supplement Figure S1A), whereas reduced lyso-PC species comprised FAs with short (C06:0, C10:0) or mid chain length (C16:1, C18:0, C20:4). This result could be also found after normalization to total lyso-PC content. The effect of acitretin treatment on the total amount of the analyzed lyso-PC species is shown in supplementary Figure S7. Lyso-PC with short chain FAs (C06:0 and C10:0) were decreased and mainly lyso-PC species containing long chain FAs were increased (C22:0, C24:0, C26:0, C26:1, C28:0, C28:1) (supplement Figure S1C). In liver tissue, acitretin treatment resulted in 15 decreased lyso-PC species and seven increased lyso-PC species (supplement Figure S1B). Similar to brain tissue, the observed changes did not reach significance (supplement Figure S1E and S1F). However, interestingly, lyso-PC species that were found to be increased in brain, showed a trend to be decreased in liver (lyso-PC C22:0, C26:1 and C28:1) whereas lyso-PC species that were reduced in brain were found to be elevated in liver (lyso-PC 06:0, C18:0). Notably, the lyso-PC containing C18:0 fatty acid was significantly increased in liver tissue of acitretin-treated 5xFAD mice. Similar results were found by normalization to total lyso-PC content (supplement Figure S1D). Based on these findings and the fact that lyso-PC species represent the transport form of lipids^40^ one might speculate that the liver provides lyso-PC species that are lacking in brain after acitretin treatment.

A decrease in lyso-PC is closely linked to a reduced PLA2 activity, indicating that acitretin might impair PLA2 activity. Taken into consideration that PLA2 is increased in AD^41,42^, the potential acitretin-induced reduction in PLA2 activity might be, beside increasing plasmalogen levels as mentioned above, another beneficial property of acitretin in respect to AD treatment.

## Effect of acitretin on SM species in 5xFAD transgenic mice

**Figure 6:**
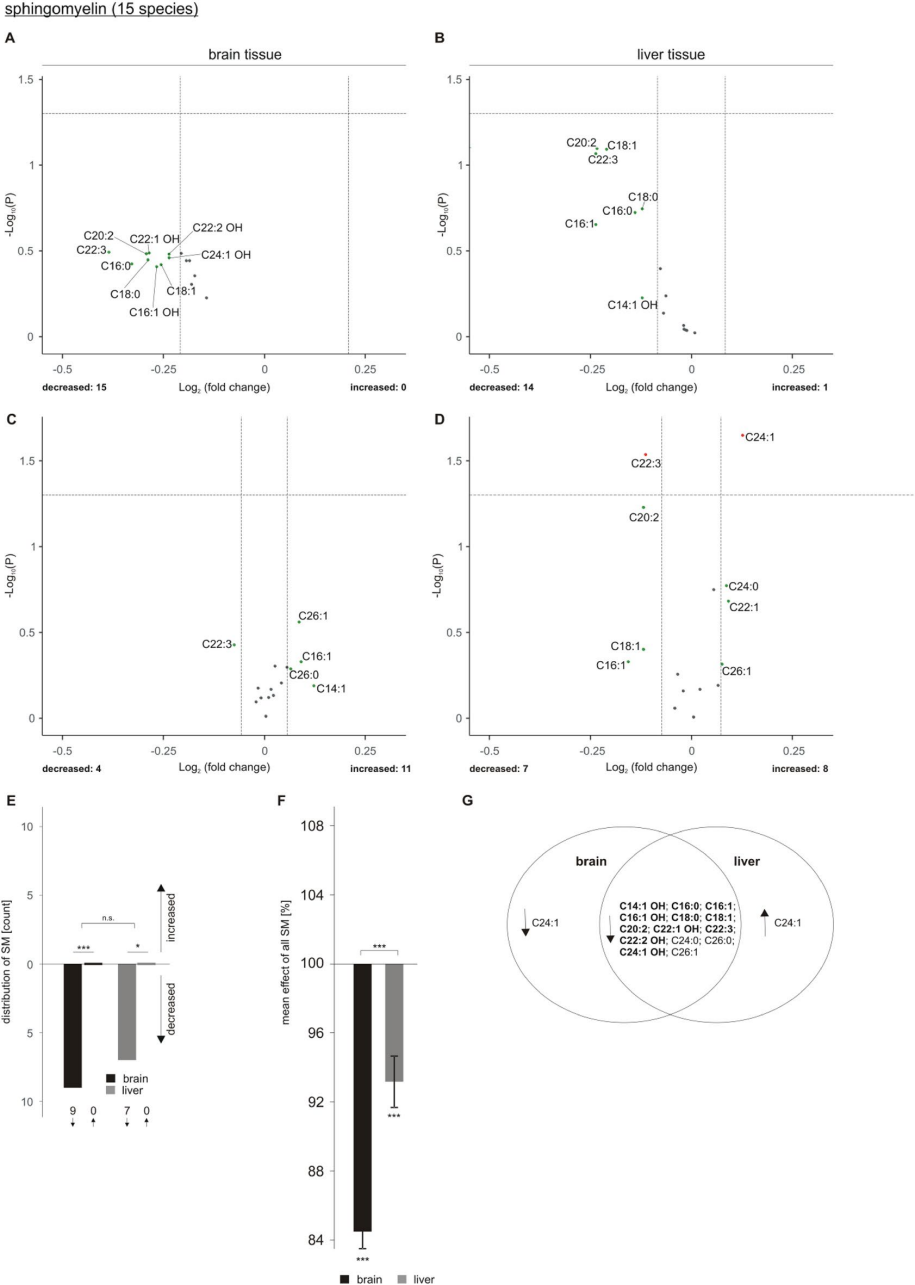
Changed sphingomyelin (SM) levels in 5xFAD brain and liver tissue after acitretin treatment. Fold changes of single SM species in brain and liver tissue are shown as volcano plots at the top (**A**: brain tissue, **B**: liver tissue) and the effects of single species independent of lipid class effect for the analyzed species are presented as appropriate volcano plot at the bottom (**C**: brain tissue, **D**: liver tissue). Structure and labeling of the volcano plots are according to Fig. 2. (**E**) Distribution of SM species represented as number of de- and increased parameters in brain and liver tissue in a bar chart. (**F**) Mean effects on all SM in brain and liver tissue are shown in a bar chart. Statistical significance for (**E**) and (**F**) was calculated according to Fig. 3. (**G**) Venn diagram showing exclusively changed as well as overlapping SM species in brain and liver tissue. Species with a fold change greater than the SEM are highlighted in bold.

The analysis of 15 SM species in brain tissue of acitretin-treated AD model mice compared to the control group revealed that all 15 analyzed SM species tended to decrease (Fig. 6A). Also for liver tissue we found 14 out of 15 SM species to be decreased (Fig. 6B). For both tissues, the shift towards decreased SM level and the number of downregulated SM species was significant (Fig. 6E,F), with an even more pronounced effect in the brain. The observed reduction in SM species upon acitretin treatment might be critical in respect to AD treatment as SMases, the catabolic enzymes for sphingomyelines, are reported to be increased by Aβ or in AD brain^43,44^ and acitretin might aggravate the effect on SM level.

Beside the reduction in SM levels we found two SM species to be significantly altered in liver tissue when normalized to total SM content: SM C24:1 was significantly increased after acitretin treatment whereas SM C22:3 was significantly decreased (Fig. 6D). For brain tissue no significant alterations were detected (Fig. 6C). The effect of acitretin treatment on the total amount of the analyzed SM species is shown in supplementary Figure S7. The Venn diagram shows overlapping species and those who are de- or increased in brain or liver tissue exclusively (Fig. 6G).

## Acitretin-induced changes of carnitine species in brain and liver tissue of AD transgenic mice

**Figure 7:**
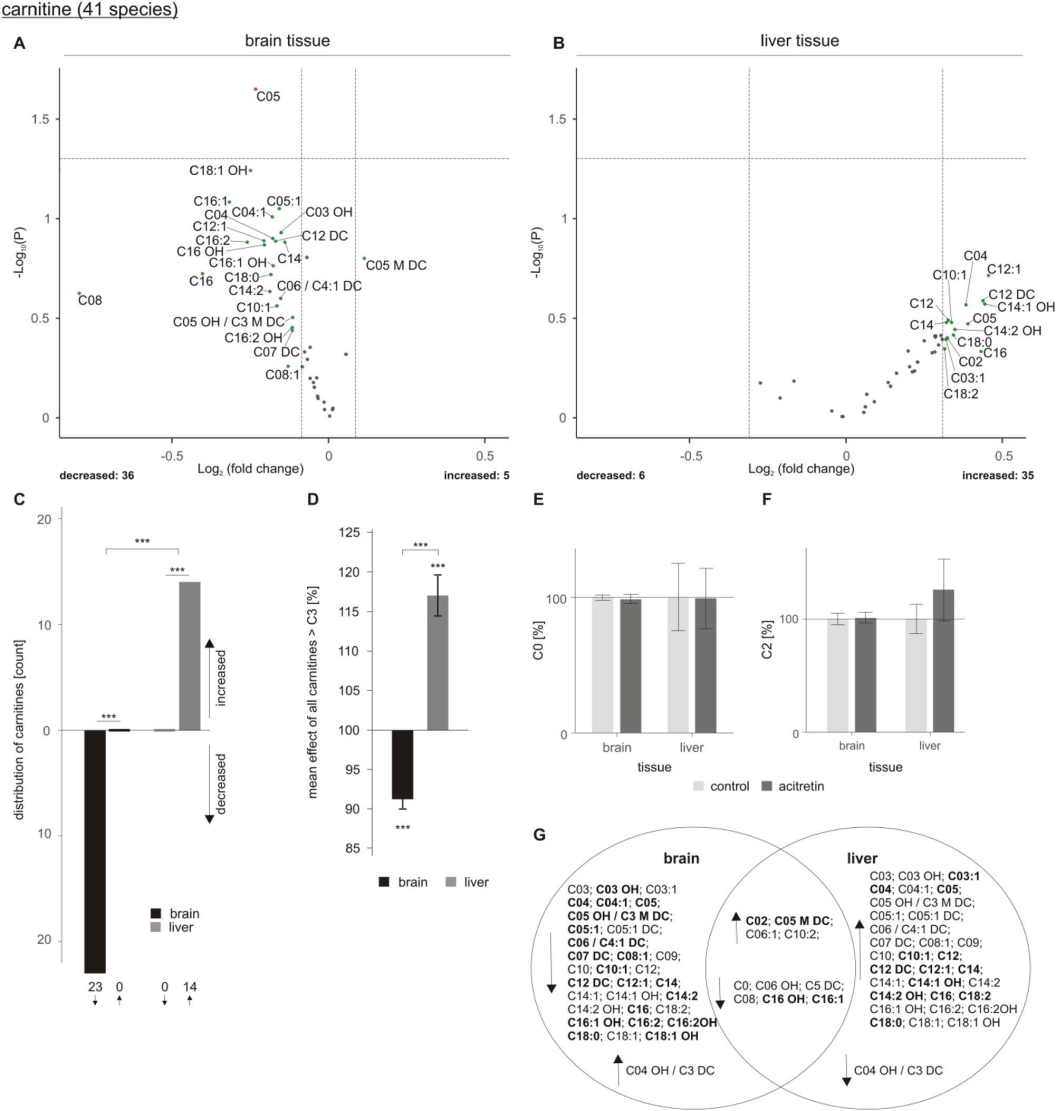
Changed carnitine levels in 5xFAD brain and liver tissue after acitretin treatment. Fold changes of single carnitine species in brain and liver tissue are shown as volcano plots at the top (**A**: brain tissue, **B**: liver tissue). Structure and labeling of the volcano plots are according to Fig. 2. (**C**) Distribution of carnitine species represented as number of de- and increased parameters in brain and liver tissue in a bar chart. (**D**) Mean effects on all carnitines in brain and liver tissue are shown in a bar chart. Statistical significance for (**C**) and (**D**) was calculated according to Fig. 3. Fold changes of carnitines CO (**E**) and C2 (**F**) in brain or liver tissue after acitretin treatment are represented as bar chart. Statistical significance was calculated using two sample t-test. (**G**) Venn diagram showing exclusively changed as well as overlapping carnitine species in brain and liver tissue. Species with a fold change greater than the SEM are highlighted in bold.

In line with reduced TAG and PCaa level in brain tissue of acitretin-treated transgenic animals, we found that 36 out of 41 measured carnitine species were decreased, whereas five revealed a small increase with an effect strength within the average SEM (Fig. 7A). For 22 carnitine species a decrease with an effect strength out of the SEM was detected and one carnitine species (C05) showed a significant reduction in transgenic mice after acitretin treatment pointing towards an effect on short/branched chain acyl CoA dehydrogenase (SBCAD), which should be addressed in further studies in detail. The observed effect was more pronounced for acyl-carnitines (CX, X > 3), indicating changes in β-oxidation. The observed acitretin-induced decrease in carnitine species in brain tissue was highly significant (Fig. 7C). In contrast to the downregulated carnitine metabolism in brain tissue of acitretin-treated AD transgenic mice, carnitine species tended to increase in liver tissue. 35 carnitine species tended to be elevated, 14 out of them revealed an increase with an effect strength higher than the average SEM (Fig. 7B). The shift towards downregulation of carnitine species in brain and the shift towards an upregulation of carnitine species in liver after acitretin treatment were highly significant (Fig. 7C). Significant alterations were also found for the mean effect of all carnitines (CX, X > 3): in brain a significant reduction was found, in liver a significant increase (Fig. 7D). Notably, for both tissues the total level of carnitine species (CX, X > 3) showed a similar trend as obtained for TAG and PCaa species. TAG, PCaa and carnitines were reduced in brain whereas TAG, PCaa and carnitine species were increased in liver. This observation can be explained by the fact that TAGs are mainly stored in lipid droplets, which are surrounded by phospholipids, in particular PCaa. Fatty acids derived from TAG are used for energy production in the mitochondria by transferring them to carnitines. However, no significant alterations were detected for carnitine (C0) and acetyl-carnitine (C2) (Fig. 7E,F). The effect of acitretin treatment on the total amount of the analyzed acylcarnitine species is shown in supplementary Figure S7. The Venn diagram shows overlapping species and those who are de- or increased in brain or liver tissue exclusively (Fig. 7G).

## Discussion

Innovative new drugs are still urgently needed in AD therapy as various clinical trials failed (for a view on current state of clinical trials:^45^) and in general the development of new psychiatric medications proceeds slowly. Acitretin showed several favorable properties with respect to pathological hallmarks of AD and was able to evoke the non-amyloidogenic α-secretase enhancing potential in a small cohort of AD patients^11^. Nevertheless, the target population in AD is found in the elderly who display several critical features regarding pharmaceutical treatment such as decreased liver blood flow and detoxification capability^46^, poly-medication and a lowered integrity of the blood–brain barrier^47^. For acitretin, there have been cautions since the beginning of its systemic use in dermatology, regarding its hepatotoxic potential and a potential increase in LDL-cholesterol and TAGs in serum. Here, we provide a detailed semi-quantitative lipidomics approach, analyzing lipid changes in brain and liver tissue of AD transgenic mice treated with acitretin. The influence of acitretin on lipid alterations in brain can be either provoked by altered lipid composition in the blood stream fed by the liver or by a direct effect of acitretin on brain lipid homeostasis as acitretin is able to cross the blood brain barrier within cell models but also in mice^48,49^.

**Figure 8:**
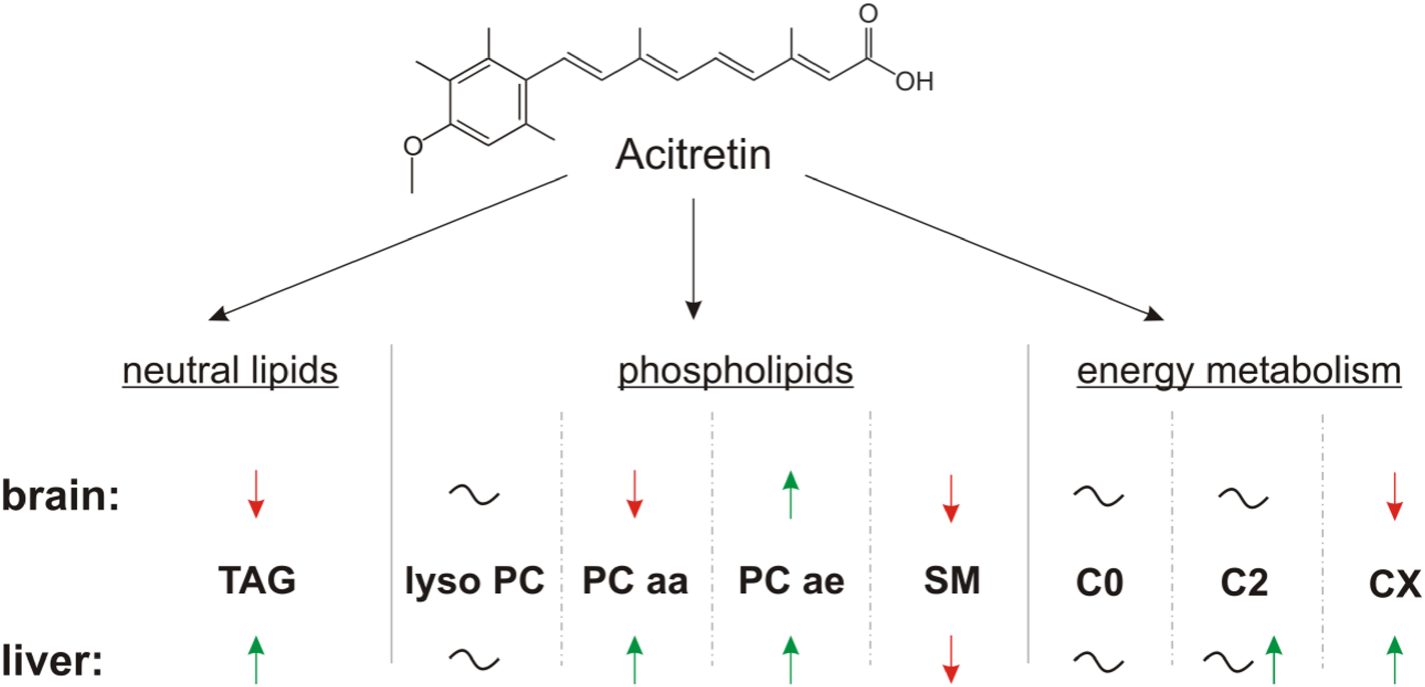
Summary of the observed effects of acitretin on the analyzed neutral lipids, phospholipids and on energy metabolism in brain and liver tissue of and 5xFAD mice. FAD: Familial Alzheimer´s Disease. FA: fatty acid. TAG: triacylglycerides. lyso-PC: lyso-phosphatidylcholine. PCaa: phosphatidylcholine. PCae: phosphatidylcholine-plasmalogen. SM: sphingomyelin.

In general, we found that some lipid classes were altered similarly in brain and liver of acitretin-treated AD transgenic mice, whereas others revealed tissue-type specific effects (Fig. 8). As already mentioned in the result section, the data obtained by mass spectrometry are semi-quantitative and the recorded alterations are shown in x-fold or % change compared to the control group. Moreover, the observed results were normalized to deuterated standards added before lipid extraction to avoid inaccuracies caused by deviations in the yields of lipid extraction or ionization efficiency. Utilizing a lipidomics approach, matrix effects, in which one lipid (class) influences the ionization or signal of other lipids, may occur. In order to estimate whether differences in the lipid composition of acitretin-treated and control mice may result in matrix effects, lipid extracts were added to a synthetic composition of deuterated lipids and the ratio between the deuterated lipid species were calculated (supplemental Figure S5B). As there were no statistical differences between the ratios of the treated and control group, we assume that matrix effects play a limited role in our analysis (supplemental Figure S5B). However, for single species the impact of matrix effects cannot be ruled out completely. Moreover, it has to be pointed out that by analyzing more than 700 parameters, some results might reach significance by chance. Several statistical methods exist dealing with this issue to reduce “false-positive “ results by multiparametric analysis, e.g. by performing a Bonferroni correction. On the other hand, these statistical methods, especially when performed on this amount of analyzed parameters, lead to a drastically reduction of significantly altered parameters, resulting in an increased possibility of “false-negative “ or unnoticed parameters. To address this problem, we decided to show our results as Volcano plots, combining effect strength and significance, in general and for each lipid class. Although many parameters did not reach significance, it becomes obvious by this illustration that some lipids within lipid classes behave similar and that single lipid species, although being not significantly altered, show comparable effects. For example, in liver tissue of acitretin-treated 5xFAD mice, 40 PCaa species showed an increase whereas only three PCaa species were found to be decreased. 23 out of these PCaa species had an effect magnitude higher than the average SEM but did not reach a significant level. Taken into consideration that “false-positive “ effects occur, one would assume that a decrease and an increase of the analyzed parameters would appear in an equal or comparable amount. In line with this argumentation, we would like to emphasize the importance of analyzing lipid species with common chemical properties as a cluster. In our study we focused on the head group, the ester or ether bond, the saturation, and the chain length as parameters for chemical similarities between the lipid species.

Furthermore, we compared the observed effects of the two different tissues, brain and liver of acitretin-treated 5xFAD mice, in order to elucidate similarities or differences between the types of tissue. We found that plasmalogens, lyso-PC and SM species behave similar in both tissues whereas differences were observed for TAG, PCaa and carnitines. Most pronounced, TAGs were elevated in liver tissue of transgenic mice treated with acitretin, which is in line with findings that acitretin treatment is associated with the prevalence of nonalcoholic fatty liver disease in patients suffering from psoriasis^29,30^ and that TAG are increased in serum/blood of acitretin-treated patients^13,14^. Opposed to this, for brain tissue most TAG species tended to decrease, revealing a significant reduction in the mean effect of all analyzed TAG species. The opposite effect of acitretin on TAG species in brain compared to liver tissue might be related to the findings of decreased glucose metabolism in specific brain areas of AD patients and AD transgenic mice^50–52^. As glucose metabolism is impaired in AD-affected brains, the organism might compensate the reduced energy homeostasis by an increased β-oxidation. However, it has to be underlined that this kind of compensation might be more pronounced in the early stages of the disease, as a mitochondrial dysfunction in severe AD patients has been reported^53,54^. As a consequence, TAGs are more frequently metabolized for energy generation in the brain and this effect is elevated by acitretin-treatment. The reduced acyl-carnitines found in this study in acitretin-treated brain tissue of 5xFAD mice might also argue for an elevated β-oxidation in presence of acitretin. However, in this context it has to be mentioned that the observed reduction of acyl-carnitines in brain can also be a consequence of the reduced TAG species. In line with this argumentation, we found increased acyl-carnitine species for liver tissue of acitretin-treated transgenic animals, probably caused by the increase in TAGs. As TAGs are stored in lipid droplets, it is not astonishing that the mean effect of all analyzed PCaa species was significantly increased in liver upon acitretin treatment: phospholipids are needed to build the membranes of the lipid droplets. Vice versa, PCaa species were found to be decreased in brain tissue of acitretin-treated AD transgenic mice. Despite the different effects of acitretin on TAG and PCaa level in brain and liver, single TAG species were affected analogous in brain and liver. Normalized to the total TAG content, we found that PUFA-containing TAGs (TAG C56:8, C58:5, C60:5, C54:9) were reduced in brain and liver. These TAG species might contain DHA (C22:6) and EPA (C20:5), which are discussed to be reduced in brains of AD patients^55,56^ and which are closely linked with AD pathology^57–63^. As this result points to a direction that acitretin treatment leads to a further reduction in the omega-3 FAs DHA and EPA, supplementation of PUFAs in particular of DHA and EPA in AD patients treated with acitretin should be taken into consideration.

A further aspect that has to be considered is a potential SM-reducing property of acitretin, which was observed in brain and liver tissue. SM levels have been reported to be decreased in AD brains, probably caused by the Aβ-induced activation of sphingomyelinases degrading SM to ceramide^43,44^. Interestingly, sphingomyelinase inhibitors are discussed to treat diseases associated with enhanced activity of acid sphingomyelinase, e.g. major depression and AD. Remarkably, acitretin treatment is reported to be associated with depression and suicidal ideation^64–67^, which might be explained by our finding that acitretin decreases SM level.

Beside these caveats, we also found beneficial properties of acitretin in respect to changes in lipid classes associated with AD. Notably, plasmalogens that have been found to be significantly reduced in *post mortem* brain samples and cerebrospinal fluid of AD patients^68–70^ showed an elevated level after acitretin treatment in brain and liver of transgenic animals. Besides being important structural lipids in cell membranes, plasmalogens are involved in many cellular processes such as membrane fusion and transport, cholesterol efflux, diffusion of signal-transduction molecules, membrane-bound enzyme activity and have antioxidant properties. In this context it has to be mentioned that the increase in plasmalogens upon acitretin treatment might have, beside their protective role against oxidative stress that is closely linked to AD, another beneficial aspect in respect to AD pathology. Plasmalogens have been found to decrease the activity of the membrane-tethered γ-secretase which is responsible for the release of Aβ in cell culture studies and in samples of human AD *post mortem* brains^70^.

Regarding lyso-PC species, we found that most of the analyzed lyso-PC species tended to decrease in brain and liver tissue upon acitretin treatment. As lyso-PC species have been reported to be decreased^24,71–73^ in AD brain one might assume that acitretin aggravates this effect on lyso-PC. On the other hand activity of phospholipase A2, an enzyme highly expressed in different brain regions, is increased in AD, per se leading to elevated lyso-PC. However, it has to be noticed that the mean effect of acitretin on lyso-PC species was not significant, indicating the need to address this aspect of acitretin on lyso-PC changes in further, particularly human studies to exactly clarify these effects. Analyzing the lyso-PC species normalized to total lyso-PC content in brain after acitretin treatment we observed lyso-PC C24:0, C26:0, C28:0 and C28:1 to be elevated. These lyso-PC species have been found to be significantly reduced in frontal cortices of AD patients^24^, indicating that acitretin treatment could eventually compensate this decline in brains of AD affected individuals.

In summary, we show that acitretin treatment exerts some positive properties in respect to lipid classes associated with AD, but also some unfavorable aspects such as decreased SM level and a decrease in DHA and EPA containing lipids. These observations have to be taken into consideration with respect to the use of acitretin in AD therapy and further studies addressing this point are indispensable. Another caveat is given by reports that APP-dependent lipid regulation differs in the pathological situation of AD and that the pathological stage of the disease might also influence or interfere with acitretin-mediated lipid alterations.

To further clarify this aspect, we have analyzed the most prominent changes of lipid species found in 5xFAD mice in a non-transgenic wild type strain in an initial experiment (C57Bl6/J, background strain of 5xFAD mice; see supplemental Figure S8). In line with our assumption that AD pathology modulates or interfere with the effect of acitretin on lipid homeostasis, we found that several changes in lipid level were not comparable in wild type mice and the transgenic mouse model. However, it has to be pointed out, that further experiments are needed to investigate the effect of acitretin in wild type mice in particular as these mice had a different age (six weeks) in our initial experiment as the transgenic mice (30 weeks), which might also have an impact on the results.

Furthermore, this lipidomics approach emphasizes the importance of lipid monitoring in patients treated with other drugs, known to influence lipid homeostasis, e.g. glucocorticoids and cyclosporine^74–77^, or in patients already being at a high risk for cardiovascular diseases or fatty liver disease. The acitretin-induced alterations in lipid classes might be closely linked to the fact that both, Aβ and the intracellular APP fragment AICD, released by amyloidogenic APP processing, are known to interfere with lipid homeostasis (summarized in supplement Figure S2)^39,43,44,78–81^ and that acitretin shifts APP processing to the non-amyloidogenic pathway. This might explain why some effects are aggravated but also some changes in lipid homeostasis seem to be attenuated. It has to be considered that we here analyzed lipid composition in females, which mostly display a stronger pathology than males (e.g. Bundy et al.^82^). If the observed phenomena are comparable in males and if they depend on disease progression will have to be investigated in future.

This lipidomics approach further underlines that lipid changes that are observed in a specific tissue or in serum after drug treatment cannot be routinely extrapolated to brain as we observed partly different and even contrary effects of acitretin in brain and liver. Metabolic conversion, drug concentration or elimination might contribute to these differences. For example, a slower elimination of acitretin from brain than from blood was found after acute injection in mice^49^.

In addition, the findings of our lipidomics approach might explain side effects of acitretin treatment, e.g. the development of non-alcoholic fatty liver disease in patients suffering from psoriasis and treated with acitretin, the association of acitretin with depression and the hepatotoxic property of acitretin as we found the inflammation-inducing arachidonic acid containing phospholipids PCaa 40:5 and PCaa 42:4 to be elevated in liver upon acitretin treatment.

## Methods

### Treatment of 5xFAD mice with acitretin

5xFAD mice (Jackson Laboratory;^83^) were stably cross-bred with C57Bl6/J mice from the animal facility of the University Medical Center of Mainz for maintenance. Transgenic female animals were used at an age of 30 weeks as indicated. Animals were housed in groups of 2–5 with free access to food and water. A 12 h light–dark cycle (6 am to 6 pm light on) was maintained at a temperature of 22 °C and a relative humidity of 60%. No specific inclusion or exclusion criteria were defined and no animals excluded from the study. A specialized randomization strategy was not used but animals were alternatingly assigned to treatment in the order of ear numbering within one cage. Experimenters were not blinded during drug injection but personnel dissecting brain and liver was blinded towards the treatment. All experiments including animals were carried out in compliance with the ARRIVE guidelines (http://www.nc3rs .org.uk/page.asp?id = 1357) and all experimental procedures were carried out in accordance with the European Communities Council Directive regarding care and use of animals for experimental procedures and were approved by local authorities (LUA Rhineland-Palatinate; G14-1-087). Acitretin was freshly dissolved in corn oil (Merck) to a concentration of 1 mg/ml. Mice were weighed on the first day of injection and the appropriate injection volume for a daily dosage of 10 mg/kg acitretin was adjusted to a total volume of 400 µl with corn oil. Mice were injected intraperitoneally for seven days including a two-day break (dos Santos Guilherme 2020^7^). Dosage was calculated according to Reagan-Shaw et al.^84^ by using the maximal dosage for human patients (50 mg/day) and a body weight for the human of 60 kg. Control animals received corn oil (400 µl). Animals were sacrificed after isoflurane anesthesia and brains and liver dissected. The left hemisphere and the right liver lobe were washed with distilled water and immediately stored at − 20 °C (for long-term storage at − 80 °C). For all further mass spectrometry experiments, homogenates of the complete left hemisphere or the right liver lobe, respectively, were used. For the exemplary analysis of the most affected lipid species within 5xFAD mice in wild type mice, C57Bl6/J female mice aged six weeks, were used.

### Measurement of different lipid species using mass spectrometry

#### Chemicals, reagents, and standards

High performance liquid chromatography (HPLC)-grade water, ethanol, and methanol were purchased from Fisher Scientific (Schwerte, Germany). HPLC-grade pyridine, phenyl isothiocyanate (PITC) and ammonium acetate were acquired from Merck (Darmstadt, Germany). The following standards from Avanti Polar Lipids were used for normalization: 06:0 PC (DHPC), 19:0 Lyso PC, 08:0 PE, 06:0 SM (d18:1/6:0), and Splash II Lipidomix Mass Spec Internal Standard. The carnitine standards octanoyl-L-carnitine d3 and palmitoyl-L-carnitine d3 were purchased from Supelco Analytical.

#### Sample preparation

Mouse brain and liver samples were mechanically homogenized in water via Minilys (PEQLAB, Erlangen, Germany) for 60 s on maximum intensity. Protein was measured using bicinchoninic acid assay according to Smith et al.^85^ and homogenates were adjusted to a protein amount of 10 mg/ml in water.

#### Lipid extraction

The used solid/liquid lipid extraction method is described in detail in Grimm et al.^24^. Briefly, a 96 well filter plate (0.45 μm; Merck) was fixed on a 96-deep well plate (Fisher Scientific) and circles of whatman blotting paper with a diameter of 6 mm were placed into the wells of the filter plate. On these Whatman papers a standard mixture was added, followed by 10 μl of each prepared sample (described above). After drying the samples under a nitrogen flow (1–2 bar) for 45 min, 20 μl of 5% PITC (v/v) diluted in ethanol / water / pyridine (1:1:1, v/v/v) were added to the wells and incubated for 20 min at room temperature. Samples were again dried for 45 min under nitrogen, before lipids were extracted by the use of 300 μl 4.93 mM ammonium acetate in methanol and shaking the plate for 30 min at 450 rpm on a plate shaker (IKA, Staufen, Germany). Liquid samples were transferred into the 96-deep well plate by centrifugation for 2 min at 500×g. Afterwards, the samples were diluted with 600 μl 5 mM ammonium acetate in methanol/water (97:3, v/v), the plate was covered with a silicone mat and shaken for further 2 min at 450 rpm and room temperature before mass spectrometry analysis. An average extraction efficiency of > 80.7% (intra-day variance of 3.9%) and a linearity of R^2^ > 0.96 for this lipid extraction method were determined for these experimental conditions (see supplemental Figure S5D).

#### Mass spectrometry

For measurement of different species of diacyl-phosphatidylcholines (PC aa), phosphatidylcholine-plasmalogens (PC ae), lyso-phosphatidylcholines (Lyso PC), acyl- and acetyl-carnitines, sphingomyelins (SM) and triglycerides (TAG) a 4000-quadrupole linear-ion trap (QTrap) equipped with a Turbo Spray ion source (AB Sciex, Darmstadt, Germany) was used. Detection of different lipid species was carried out in triplicates using the Analyst 1.4.2 software (AB Sciex, Darmstadt, Germany) with help of an autosampler of the Agilent HPLC 1200. Lipid analysis was performed in positive mode using the following parameters: measurement period = 3 min, scan type = multiple reaction monitoring (MRM), curtain gas = 20.0 psi, collision gas = medium, ion spray voltage = 5500.0 V, temperature = 200.0 °C, ion source gas 1 = 40 psi, ion source gas 2 = 50 psi, interface heater = on, entrance potential = 10 V, collision cell exit potential = 15 V. The used Q1 and Q3 masses, declustering potentials (DP) and collision energies (CE) for each metabolite were derived, among others, from^24,86^ and are listed in supplementary table 1. The constantly measured intra- and inter-day variance was in average 6.5% (see supplemental Figure S5A). Potential matrix effects were evaluated by calculating the ratio between the deuterated lipid standards in presence of lipid extracts from acitretin-treated and control mice. The change in the ratio was maximum 3.2% and in average 1.4% as shown in supplemental Figure S5B.

### Oil red O staining of liver tissue

Liver tissue from long-term storage at − 80 °C was embedded into Tissue Freezing Medium (Leica #14020108926) and immediately frozen at − 80 °C. 14 µm sections were prepared at − 20 °C, collected on glass slides and stored at − 80 °C. For the Oil Red O (ORO) staining, the slides were incubated with 60% (v/v) isopropanol for five minutes, followed by 10 min incubation with fresh ORO working solution (7:5 dilution of ORO stock solution with water; ORO stock solution: 300 mg ORO in 100 ml 100% isopropanol). Destaining was performed by two consecutive washing steps for two minutes in 60% isopropanol and with 100 rpm shaking, respectively. After three washing steps of one minute each in water, slides were mounted with Fluormount G onto coverslips. For visualization, slides were scanned using a ZEISS Axio Scan Z1 (supported by the DFG INST 256/434-1 FUGG) with 20× magnification. A maximum intensity projection of three 1 µm z-sections is shown.

### Data analysis and statistical analysis

Counts per second for each MRM pair were extracted via the Analyst 1.4.2 software (AB Sciex, Darmstadt, Germany). Each lipid was normalized to its respective lipid class standard. After normalization, the mean per triplicate was formed for each lipid/standard ratio per mouse (n = 10; five control mice, five acitretin-treated). In Figs. 3, 4, 5 and 6 C/D lipid species signals were divided by the sum of the lipid class of the lipid species, e.g. PC(aa) species X / total of all PC(aa) species. This normalization allows analyzing the distribution of the lipid species within a lipid class. Statistical analysis was carried out with R (R Core Team 2020; Vienna, Austria; https://www.R-project.org/). *p* value calculation for each parameter, shown in volcano plots (see supplemental table 2), was carried out using two-tailed student’s t-test. Volcano plots were created via the R package „EnhancedVolcano “ (Kevin Blighe, Sharmila Rana and Myles Lewis (2020). version 1.6.0. https://github.com/kevinblighe/EnhancedVolcano). Statistical analysis of the average lipid class effect against the respective control was carried out via a two-tailed one-sample t-test. Differences for the mean lipid class effect between tissue types were calculated via two-tailed student’s t-test. To determine whether the lipid distribution, for lipids beyond the average lipid class SEM in a tissue, is significant, we used the binomial test with a 50% likelihood of occurrence of increased lipids. To determine if the increased/decreased lipid distribution significantly differs between the tissue types we used the fisher’s exact test. Heatmaps were created via the R package pheatmap (Raivo Kolde (2019); version 1.0.12. https://CRAN.R-project.org/package=pheatmap). Images were created using CorelDRAW 11 (Corel Cooperation, Ottawa, Canada). Error bar graphs represent standard error of the mean. Significance was set at *p ≤ 0.05, **p ≤ 0.01 and ***p ≤ 0.001.

## Supplementary Information

Supplementary Information 1.

Supplementary Information 2.

## Data Availability

All data generated or analyzed during this study are included in this published article (and its Supplementary Information files).
