# Supplementary material for: Shotgun lipidomics of liver and brain tissue of Alzheimer’s disease model mice treated with acitretin
Source: Sci Rep. 2021 Jul 27;11:15301. doi: 10.1038/s41598-021-94706-3 (PMC8316403; doi:10.1038/s41598-021-94706-3)
Supplement: Supplementary file 2 — Supplementary Information 2. [file 41598_2021_94706_MOESM2_ESM.pdf]

Supplementary material

Shotgun lipidomics of liver and brain tissue of Alzheimer's disease model mice treated with acitretin

Anna A. Lauer[1#], Daniel Janitschke[1#], Malena dos Santos Guilherme[2#], Vu Thu Thuy Nguyen[2], Cornel M. Bachmann[1], Sen Qiao[3], Bianca Schrul[4], Ulrich Boehm[3], Heike S. Grimm[1], Tobias Hartmann[1,5], Kristina Endres[2§*], Marcus O.W. Grimm[1,5§*]

[1] Experimental Neurology, Saarland University, Homburg / Saar, Germany; Anna.Lauer@uks.eu; Daniel.Janitschke@uks.eu; Manuel.Bachmann@uks.eu; Heike.Grimm@gmx.de

[2] Department of Psychiatry and Psychotherapy, University Medical Center Johannes Gutenberg-University, Mainz, Germany; malenaguilherme@yahoo.de, VuThuThuy.Nguyen@unimedizin-mainz.de, Kristina.endres@unimedizin-mainz.de

[3] Experimental Pharmacology, Center for Molecular Signaling (PZMS), Saarland University School of Medicine, Homburg, Germany; sen.qiao@uks.eu; ulrich.boehm@uks.eu

[4] Medical Biochemistry and Molecular Biology, Center for Molecular Signaling (PZMS), Faculty of Medicine, Saarland University, Homburg / Saar, Germany; bianca.schrul@uks.eu

[5] Deutsches Institut für Demenzprävention (DIDP), Neurodegeneration and Neurobiology, Saarland University, Homburg/Saar, Germany; Tobias.Hartmann@uks.eu; Marcus.Grimm@uks.eu

#, § authors contributed equally to this study

* Correspondence: Kristina.Endres@unimedizin-mainz.de; Tel.: +49-6131-17-2133; Marcus.Grimm@uks.eu Tel.: +49-6841-1647927

Supplemental figure S1

lyso-phosphatidylcholine (22 species) supplement

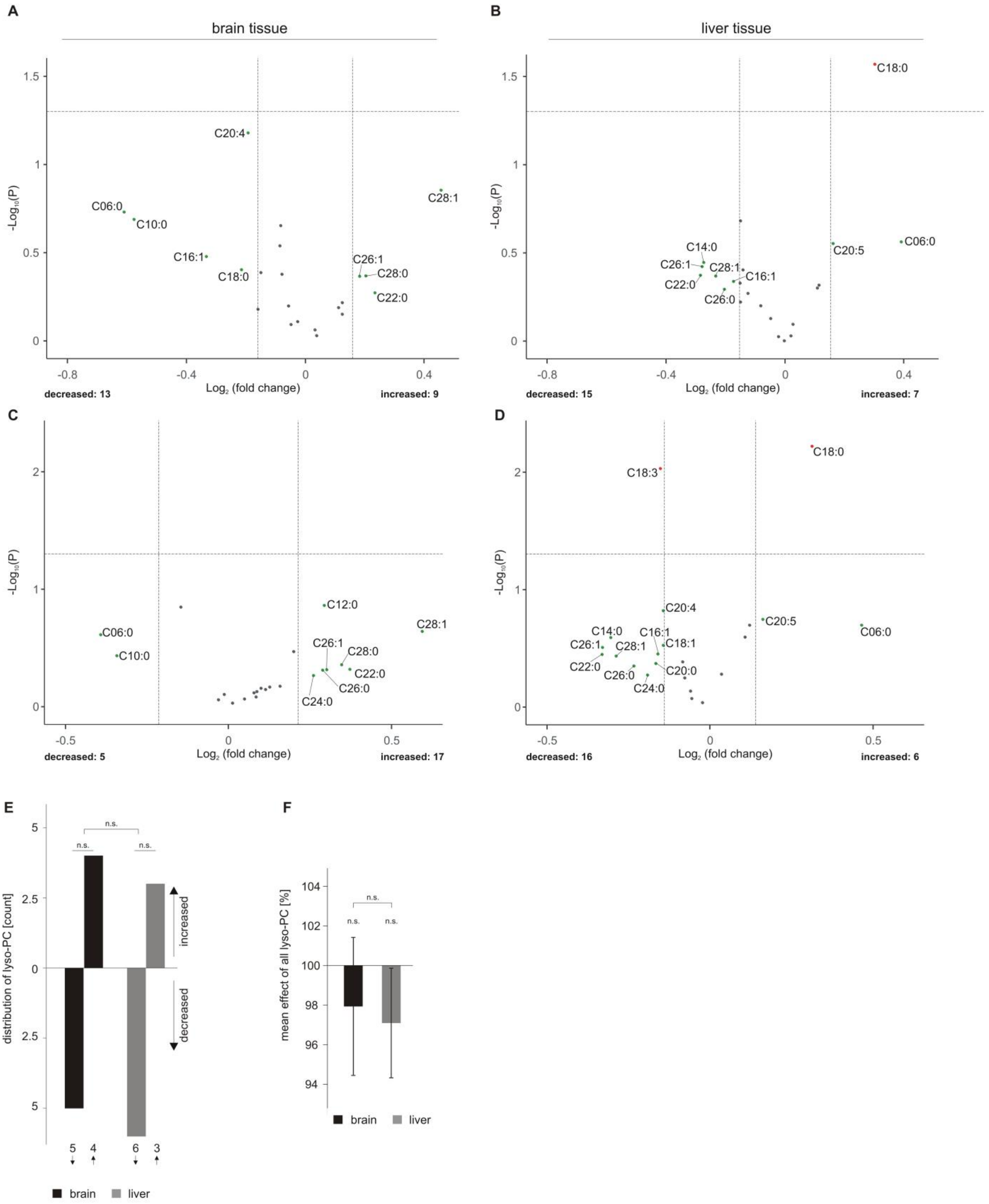

Supplemental Figure S1: Changed lyso-phosphatidylcholine (lyso-PC) levels in 5x FAD brain and liver tissue after acitretin treatment. Fold changes of single lyso-PC species in brain and liver tissue are shown as volcano plots at the top (A: brain tissue, B: liver tissue) and the effects of single species independent of lipid class effect for the analyzed species are presented as appropriate volcano plot at the bottom (C: brain tissue, D. liver tissue). Structure and labeling of the volcano plots are according to figure 2. (E) Distribution of lyso-PC species represented as number of down- and upregulated parameters in brain and liver tissue in a bar chart. (F) Mean effects on all lyso-PC in brain and liver tissue are shown in a bar chart. Statistical significance for (E) and (F) was calculated according to figure 3.

Supplemental figure S2

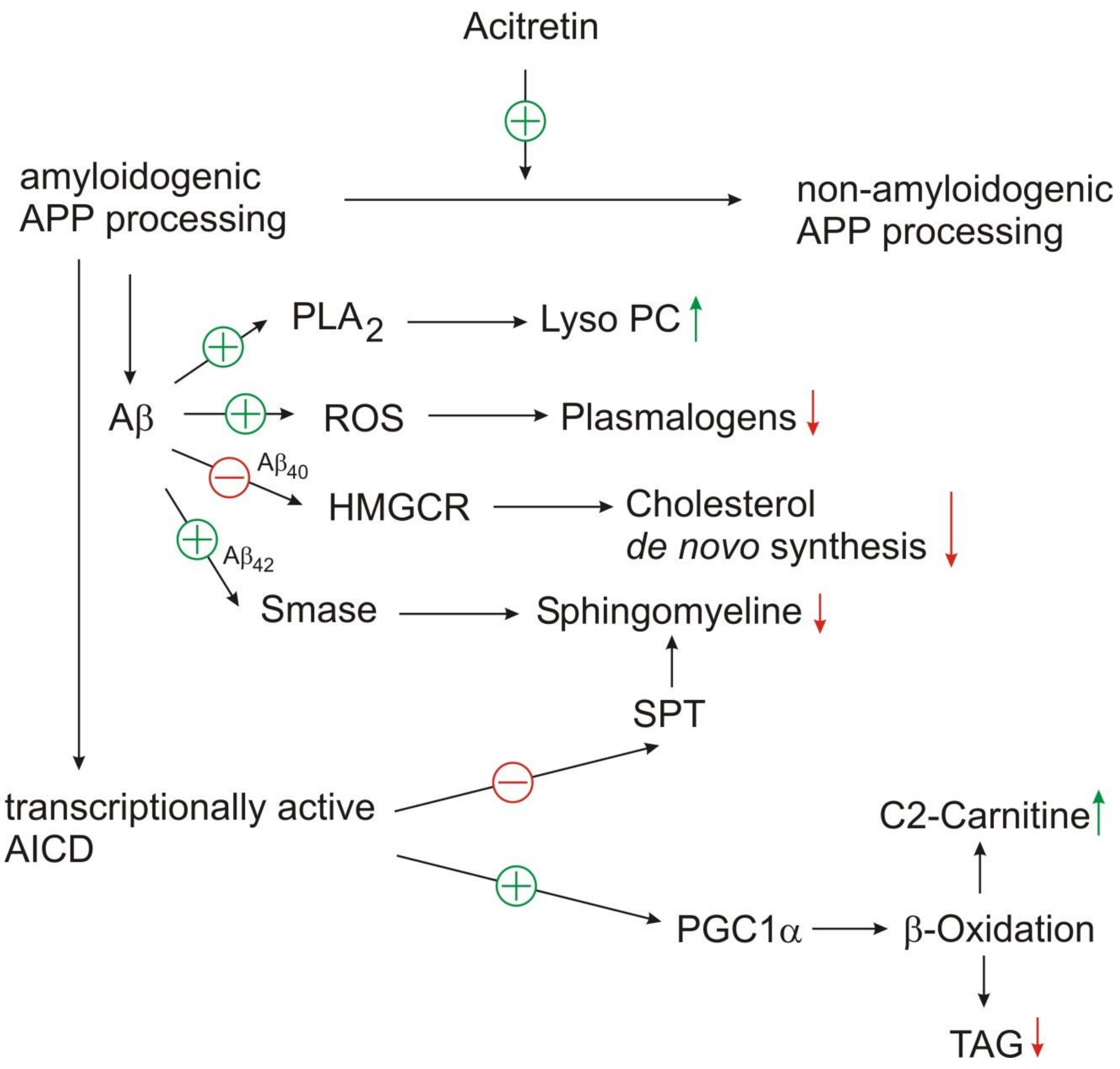

Supplemental Figure S2: Schematic overview of the influence of acitretin on processing of the amyloid-precursor protein and thereby on AD-related lipid homeostasis. PLA_2: phospholipase A2. ROS: reactive oxygen species. HMGCR: 3-hydroxy-3-methyl-glutaryl-coenzyme A reductase. SMase: Sphingomyelin phosphodiesterase. AICD: APP intracellular domain. SPT: serine palmitoyltransferase. PGC1-α: peroxisome proliferator-activated receptor gamma coactivator 1-alpha.

Figure S3

(a) saturation per lipidspecies (processed data)

Group	Lipid species	brain tissue	liver tissue
TAG	TAG SFA (X:0)	101.6% ± 8.9%	119.9% ± 19.8%
TAG	TAG PUFA (X:>3)	94.4% ± 8.3%	118.8% ± 18.1%
TAG	TAG X:1	111.7% ± 6.3%	118.1% ± 17.2%
TAG	TAG X:2	102.1% ± 9.8%	117.8% ± 16.3%
TAG	TAG X:3	103% ± 8.9%	121.5% ± 16.9%
TAG	TAG X:4	94.9% ± 7.6%	120.8% ± 18.8%
TAG	TAG X:5	89.8% ± 9.6%	114.9% ± 15.3%
TAG	TAG X:6	101.1% ± 8.2%	111% ± 18.4%
TAG	TAG X:7	101.9% ± 7.9%	109.5% ± 17.4%
TAG	TAG X:8	93% ± 10%	105.8% ± 14.7%
TAG	TAG X:9 (C54:9)	78.5% ± 9.9%	101.1% ± 21%
PC aa	PC aa PUFA	73.3% ± 10.5%	146.4% ± 40.6%
PC aa	PC aa SFA / PC aa MUFA	100.4% ± 1.6%	94.8% ± 11%
PC aa	PC aa SFA / PC aa PUFA	99.3% ± 0.2%	92.4% ± 13.4%
PC aa	PC aa MUFA / PC aa PUFA	98.8% ± 1.5%	94.3% ± 4.2%
PC aa	PC aa CX:0	72.6% ± 10.5%	111.4% ± 17.5%
PC aa	PC aa CX:1	73.3% ± 9.6%	133.8% ± 34.7%
PC aa	PC aa CX:2	75.2% ± 10.3%	156.4% ± 43.1%
PC aa	PC aa CX:3	72.8% ± 10.7%	149.9% ± 40.8%
PC aa	PC aa CX:4	71.2% ± 10.9%	137.1% ± 38%
PC aa	PC aa CX:5	73.1% ± 10.6%	139.3% ± 39.4%
PC aa	PC aa CX:6	75.2% ± 10.2%	145.5% ± 41.5%
PC aa	PC aa CX:4 / PC aa CX:6	96.3% ± 2.3%	95.8% ± 2%
PC aa	PC aa CX:4 / (CX:5 + CX:6)	96.7% ± 2.1%	98% ± 1.9%
PC ae	PC ae PUFA	108.5% ± 3.2%	105.6% ± 4.6%
PC ae	PC ae SFA / PC ae MUFA	100.6% ± 1.3%	95.2% ± 6.8%
PC ae	PC ae SFA / PC ae PUFA	100.3% ± 1.7%	95.5% ± 3.8%
PC ae	PC ae MUFA / PC ae PUFA	99.7% ± 0.7%	100.6% ± 3.7%
PC ae	PC ae CX:0	108.8% ± 3%	101% ± 3.6%
PC ae	PC ae CX:1	108.2% ± 2.8%	105.7% ± 6.8%
PC ae	PC ae CX:2	110.3% ± 4.5%	109.6% ± 5.4%
PC ae	PC ae CX:3	109.2% ± 3.1%	104.9% ± 6.7%
PC ae	PC ae CX:4	103.6% ± 1.2%	101.8% ± 4.1%
PC ae	PC ae CX:5	108.9% ± 3.1%	102.5% ± 4.3%
PC ae	PC ae CX:6	107.7% ± 3.6%	107.3% ± 5%
PC ae	PC ae CX:4 / PC ae CX:6	96.2% ± 2.2%	94.6% ± 1.6%
PC ae	PC ae CX:4 / (CX:5 + CX:6)	95.5% ± 1.8%	97.3% ± 1.2%
Lyso PC	Lyso PC PUFA	92.9% ± 3%	99.4% ± 6.9%
Lyso PC	Lyso PC SFA / Lyso PC MUFA	105.4% ± 3.7%	124.2% ± 8%(*)
Lyso PC	Lyso PC SFA / Lyso PC PUFA	94% ± 11.2%	106.8% ± 10.8%
Lyso PC	Lyso PC MUFA / Lyso PC PUFA	89.9% ± 11.7%	88.7% ± 11.3%
Lyso PC	Lyso PC CX:0	87.8% ± 10.4%	104.9% ± 3.1%
Lyso PC	Lyso PC CX:1	83.6% ± 10.5%	87.4% ± 8.3%
Lyso PC	Lyso PC X:2 (Lyso PC 18:2)	98.2% ± 3.8%	107.8% ± 8.1%
Lyso PC	Lyso PC CX:3	96.6% ± 5.8%	104.6% ± 6.2%
Lyso PC	Lyso PC X:4 (Lyso PC 20:4)	87.5% ± 4.4%	90.7% ± 6.1%
Lyso PC	Lyso PC X:5 (Lyso PC 20:5)	102.3% ± 6%	111.8% ± 7.3%
Lyso PC	Lyso PC X:6 (Lyso PC 22:6)	94.2% ± 3.2%	94.5% ± 6.7%
Lyso PC	Lyso PC C20:4 / Lyso PC C22:6	92.8% ± 5%	96.4% ± 2.7%
Lyso PC	Lyso PC CX:4 / (CX:5 + CX:6)	90.2% ± 5.8%	95.6% ± 2.7%
SM	SM SFA	82.5% ± 5.9%	93.8% ± 2.5%
SM	SM MUFA	85.8% ± 4.1%	98.6% ± 2.6%
SM	SM PUFA	77.1% ± 7%	85.3% ± 1.9%
SM	SM SFA / MUFA	97.2% ± 2.6%	94.8% ± 1.4%(*)
SM	SM SFA / PUFA	104.5% ± 2.3%	107.5% ± 1.8%
SM	SM MUFA / PUFA	106.7% ± 4.9%	113.7% ± 1.5%(**)
C	CX:0	82.3% ± 6.5%	120% ± 29.4%
C	CX:1	89.6% ± 3.5%	126.9% ± 24.6%
C	CX:2	93.6% ± 2.8%	121.4% ± 22.2%

(b) saturation per lipidspecies normalized (processed data)

Group	Lipid species	brain tissue	liver tissue
TAG	TAG SFA (X:0) / TAG total	101.9% ± 2.7%	100.5% ± 11.8%
TAG	TAG PUFA (X:>3) / TAG total	95.5% ± 1.1%	100.2% ± 5.8%
TAG	TAG X:1 / TAG total	113.3% ± 4.4%	98.5% ± 6.7%
TAG	TAG X:2 / TAG total	101.2% ± 1.6%	98.2% ± 3.3%
TAG	TAG X:3 / TAG total	102.6% ± 1.8%	101.9% ± 1.1%
TAG	TAG X:4 / TAG total	96.9% ± 1.3%	102.1% ± 6.4%
TAG	TAG X:5 / TAG total	90.1% ± 2.9%(*)	96.8% ± 4.5%
TAG	TAG X:6 / TAG total	100.8% ± 1%	92.8% ± 6.8%
TAG	TAG X:7 / TAG total	102.5% ± 2.7%	92% ± 6.4%
TAG	TAG X:8 / TAG total	94.3% ± 2.7%	90% ± 7.7%
TAG	TAG X:9 (C54:9) / TAG total	79% ± 5%	88% ± 20.9%
PC aa	PC aa PUFA / PC aa total	100.7% ± 0.7%	101.1% ± 1.2%
PC aa	PC aa CX:0 / PC aa total	100% ± 0.8%	92.7% ± 12.5%
PC aa	PC aa CX:1 / PC aa total	99.5% ± 0.8%	95.3% ± 3.2%
PC aa	PC aa CX:2 / PC aa total	102.6% ± 1.3%	110.3% ± 2%(**)
PC aa	PC aa CX:3 / PC aa total	100.7% ± 1.1%	101.6% ± 1.9%
PC aa	PC aa CX:4 / PC aa total	98.6% ± 2%	94.9% ± 0.8%(*)
PC aa	PC aa CX:5 / PC aa total	100.7% ± 1%	92.9% ± 2%
PC aa	PC aa CX:6 / PC aa total	102.4% ± 0.4%	99.2% ± 2.3%
PC ae	PC ae PUFA / PC ae total	100% ± 0.8%	101.2% ± 0.9%
PC ae	PC ae CX:0 / PC ae total	100.3% ± 1%	96.6% ± 3.5%
PC ae	PC ae CX:1 / PC ae total	99.7% ± 0.4%	101.8% ± 3.6%
PC ae	PC ae CX:2 / PC ae total	102% ± 2.2%	105.1% ± 1.7%
PC ae	PC ae CX:3 / PC ae total	100.8% ± 1%	100.8% ± 3.1%
PC ae	PC ae CX:4 / PC ae total	95.3% ± 1.5%	97.3% ± 2.9%
PC ae	PC ae CX:5 / PC ae total	100.3% ± 0.3%	98.2% ± 1.3%
PC ae	PC ae CX:6 / PC ae total	99% ± 1.1%	103.1% ± 4.4%
Lyso PC	Lyso PC PUFA / Lyso PC total	103.5% ± 9.6%	99.3% ± 4.6%
Lyso PC	Lyso PC CX:0 / Lyso PC total	100.2% ± 0.1%	104.9% ± 3.9%
Lyso PC	Lyso PC CX:1 / Lyso PC total	95.3% ± 3.1%	86.3% ± 6.5%
Lyso PC	Lyso PC CX:2 (C18:2) / Lyso PC total	109.2% ± 12.7%	107.8% ± 6%
Lyso PC	Lyso PC CX:3 / Lyso PC total	106.2% ± 13.5%	104.9% ± 3.8%
Lyso PC	Lyso PC CX:4 (C20:4) / Lyso PC total	97.9% ± 6.8%	90.5% ± 3.5%
Lyso PC	Lyso PC CX:5 (C20:5) / Lyso PC total	111.6% ± 13.6%	111.9% ± 7.6%
Lyso PC	Lyso PC CX:6 (C22:6) / Lyso PC total	105.5% ± 10.8%	94.4% ± 4.5%
SM	SM SFA / SM total	100.1% ± 0.4%	100.8% ± 1%
SM	SM MUFA / SM total	102.5% ± 2.3%	106.4% ± 0.8%(***)
SM	SM PUFA / SM total	95.3% ± 2.4%	92.9% ± 0.8%(*)
C	CX:0 / Carnitine total	96.6% ± 2.1%	99% ± 5%
C	CX:1 / Carnitine total	104.4% ± 2.8%	101.7% ± 6%
C	CX:2 / Carnitine total	108.3% ± 4.8%	95% ± 6.9%
C	CX:2 / Carnitine total	108.3% ± 4.8%	95% ± 6.9%

Supplemental Figure S3: Heat maps presenting the processed data of saturation per lipid species (A) and saturation per lipid species normalized (B).

Figure S4

(a) chain-length per lipidspecies (processed data)

(b) chain-length per lipidspecies normalized (processed data)

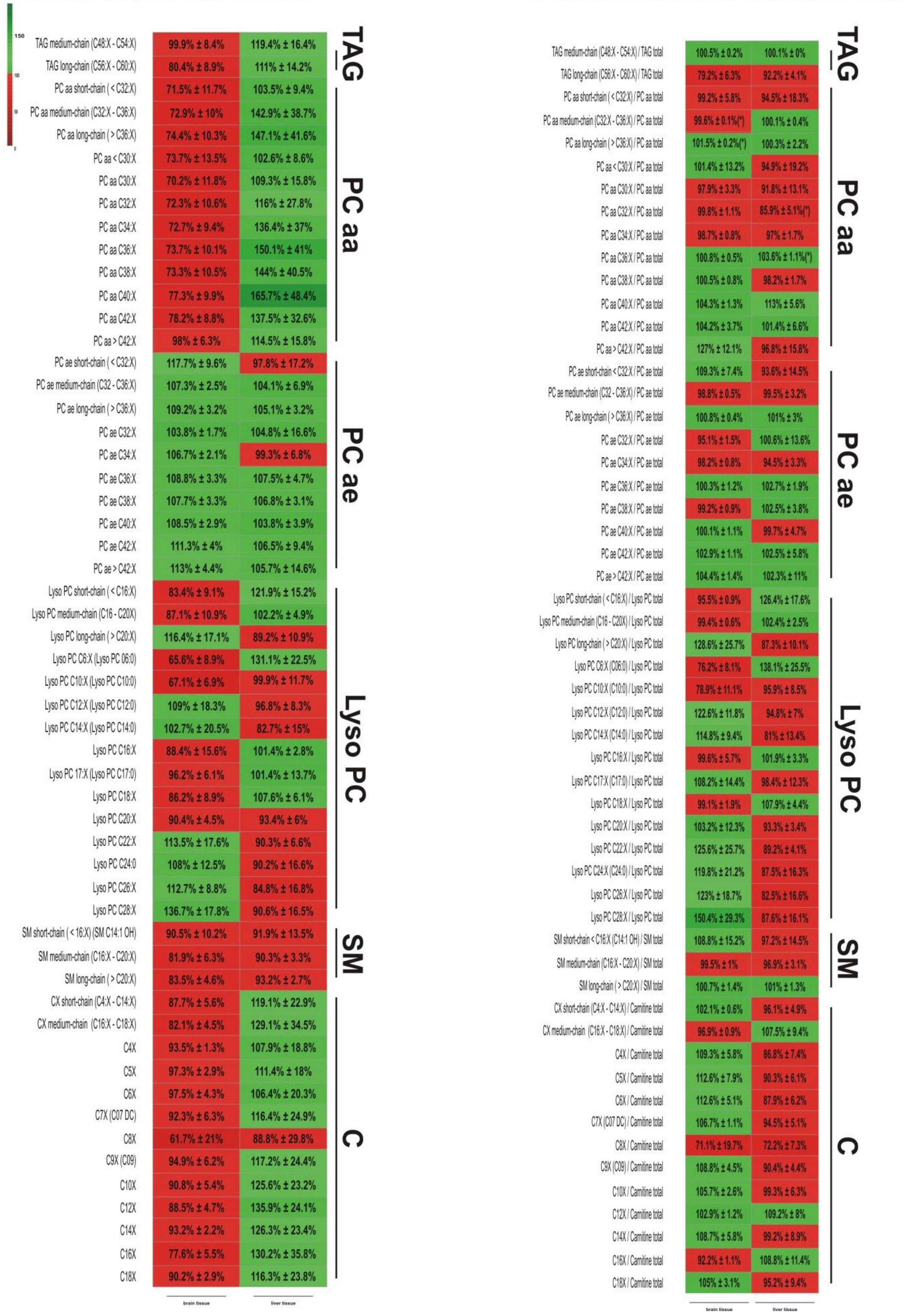

Supplemental Figure S4: Heat maps presenting the processed data of chain-length per lipid species (A) and chain-length per lipid species normalized (B).

Figure S5

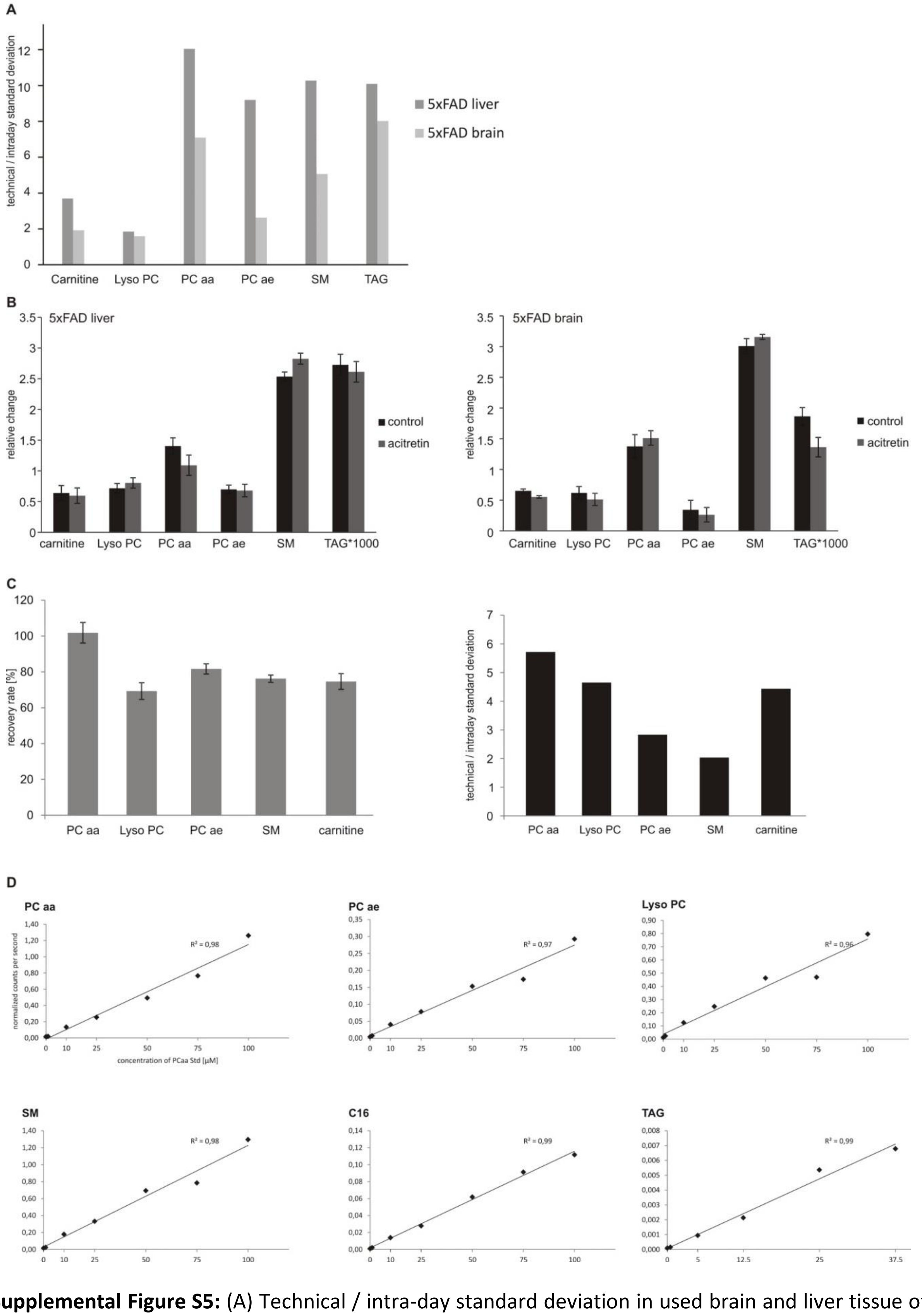

Supplemental Figure S5: (A) Technical / intra-day standard deviation in used brain and liver tissue of 5xFAD transgenic mice. (B) Calculated matrix effects of the measured parameters carnitine, lyso-PC, PCaa, PCae, SM and TAG in control and acitretin treated 5xFAD mice. (C) Recovery rate of the measured lipid species (left) and corresponding technical / intra-day standard deviation (right). (D) Linearity of the measured lipid species and corresponding correlation coefficients.

Figure S6

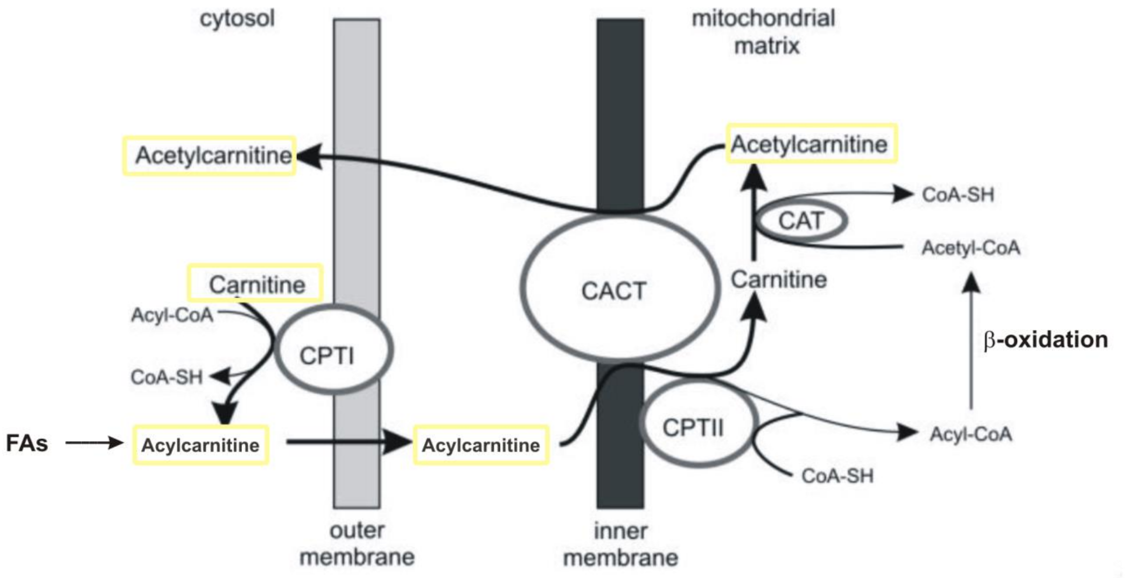

Supplemental Figure S6: Schematic overview of the transport of activated fatty acids from the cytosol to the mitochondria via the carnitine-carrier-system. Fatty acids (FA) are trans-esterified to the hydroxyl group of L-carnitine by the carnitine palmitoyltransferase I (CPT I) at the outer mitochondrial membrane. The acylcarnitine esters are further transported via the mitochondrial inner membrane by the carnitine-acylcarnitine translocase (CACT). In the mitochondrial matrix, fatty acids are trans-esterified to intra-mitochondrial Acyl-CoA by the carnitine palmitoyltransferase II (CPT II), which is a substrate for β-oxidation. The carnitine acetyltransferase (CAT) can reconvert short- and medium-chain acyl-CoA into acetylcarnitine using intra-mitochondrial carnitine. These acetylcarnitines are able to enter the cytosol via CACT.

Figure S7

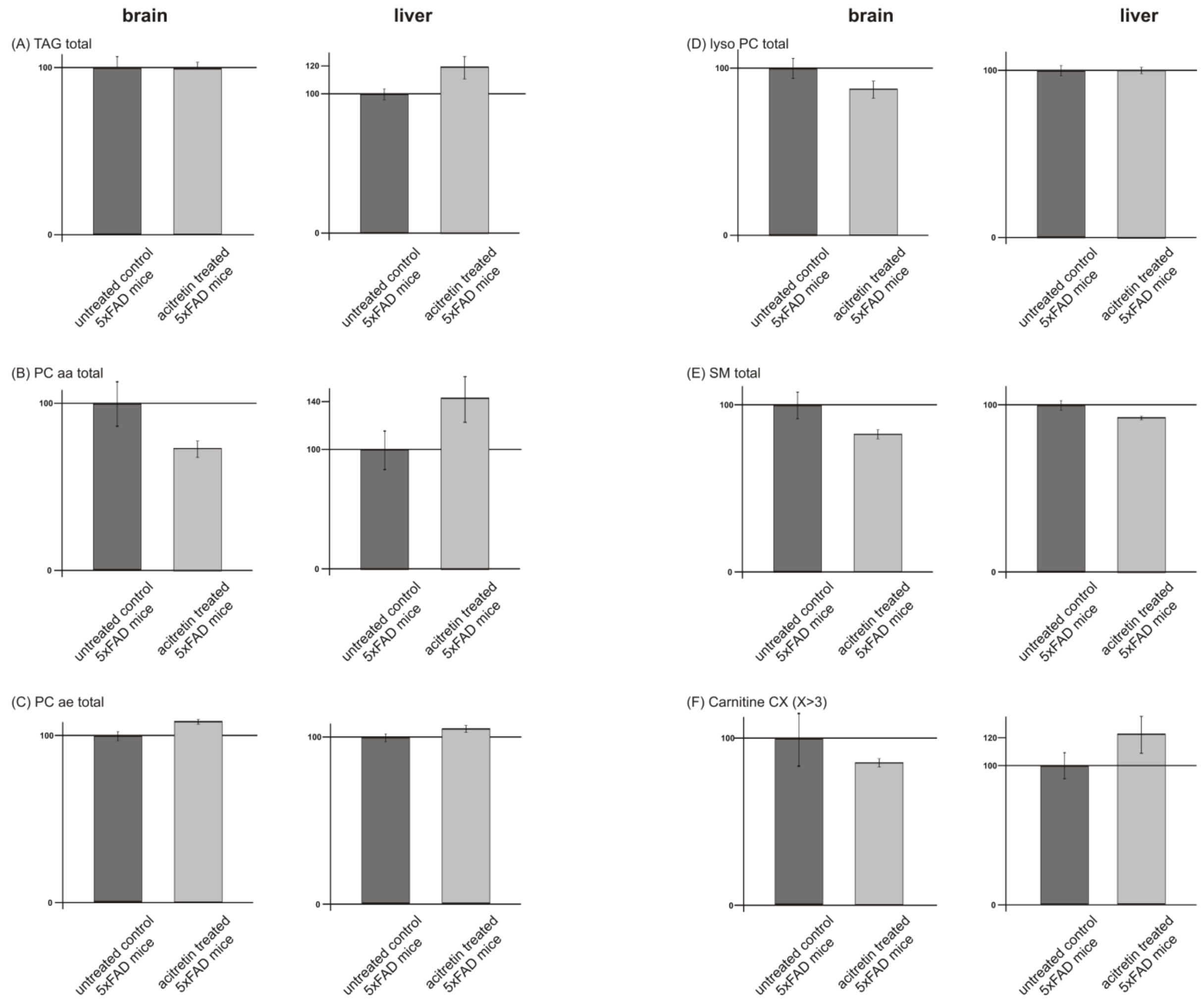

Supplemental Figure S7: Influence of acitretin treatment on the total amount of the different lipid classes. Fold changes of the total amount of each class of lipids in brain or liver tissue of acitretin-treated mice in comparison to control treated mice for triacylglycerides (A), phosphatidylcholines (B), phosphatidylcholines-plasmalogens (C), lyso-phosphatidylcholines (D), sphingomyeline (E) and carnitines (F).

Figure S8 (1)

A) triacylglycerides (39 species)

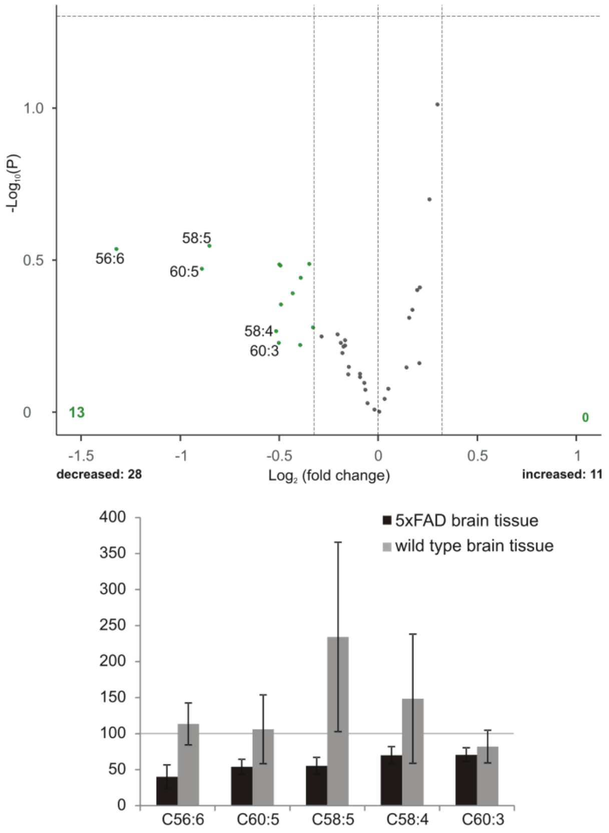

B) phosphatidylcholines (43 species)

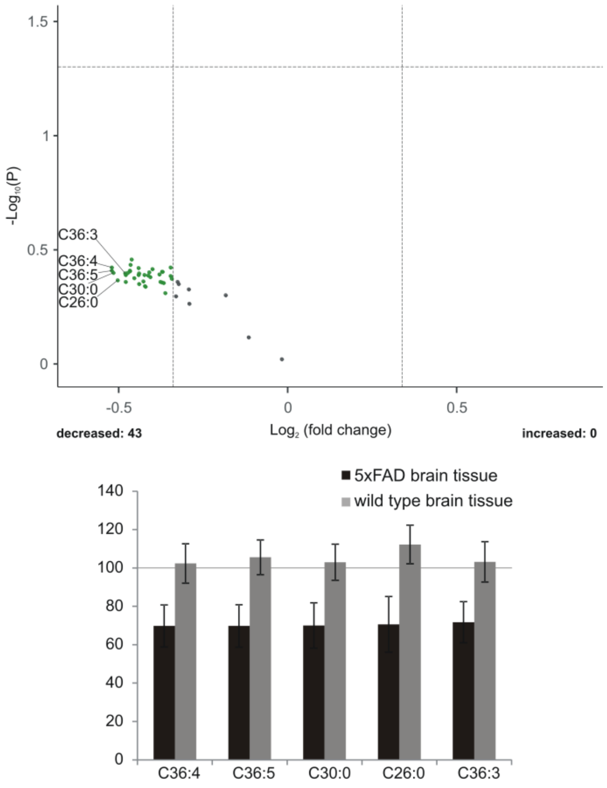

C) phosphatidylcholine-plasmalogens (39 species)

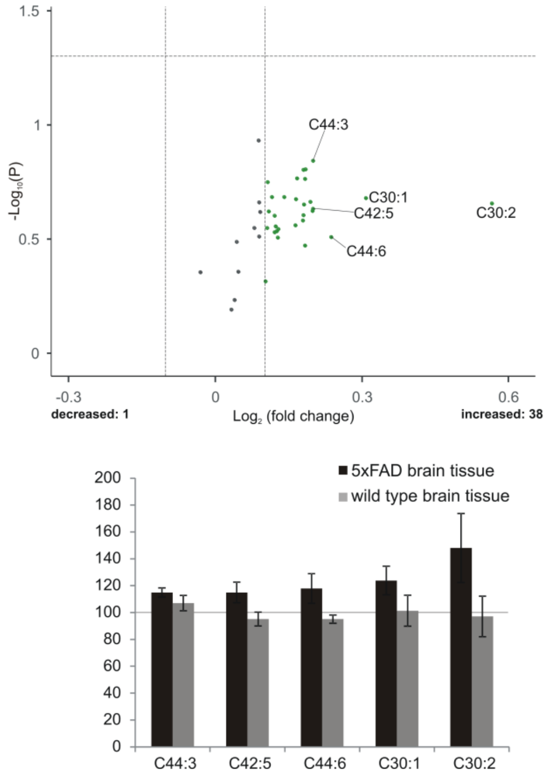

D) lyso-phosphatidylcholines (22 species)

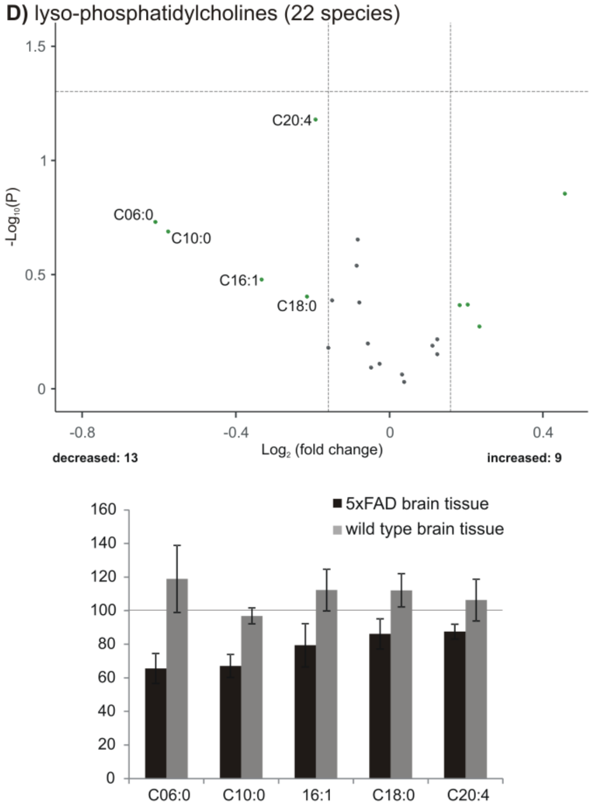

Figure S8 (2)

E) sphingomyeline (15 species)

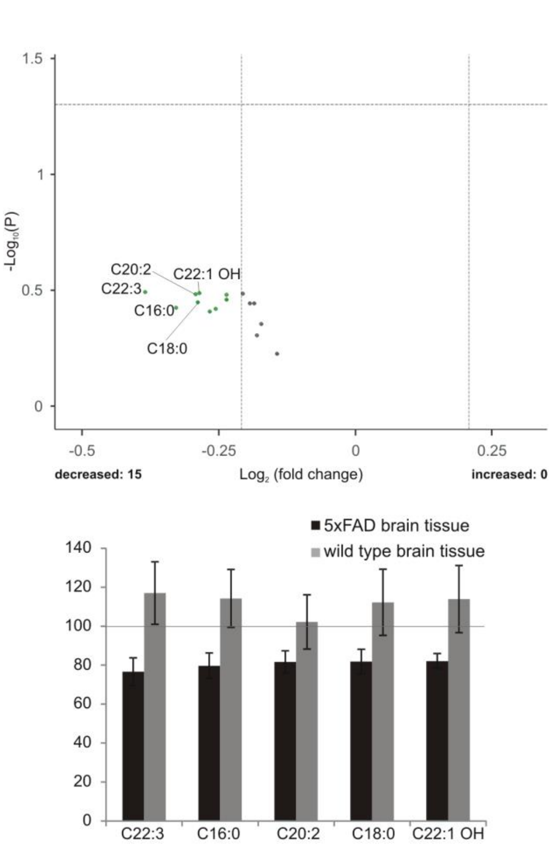

F) carnitine (41 species)

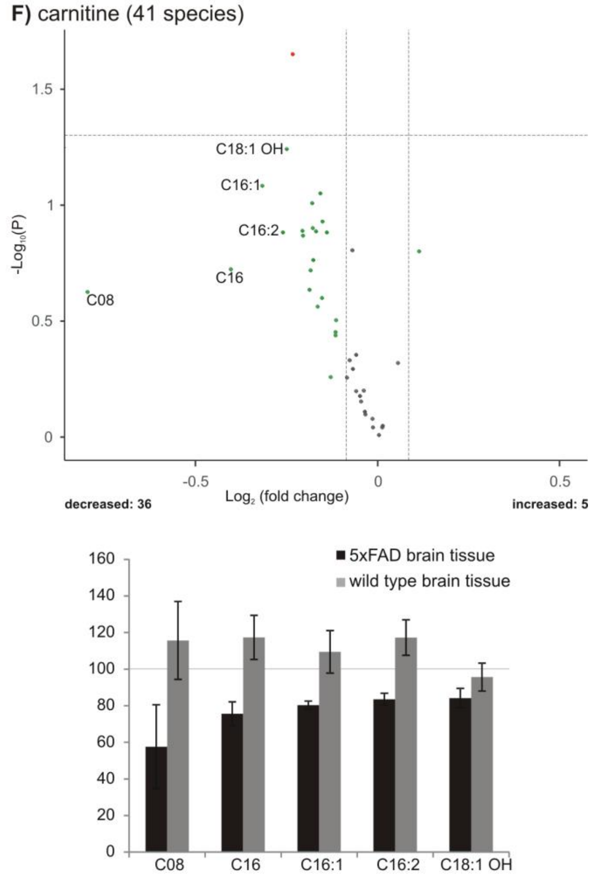

G) two-way ANOVA analysis

lipid species	p value two-way ANOVA		
	genotype	treatment	interaction
TAG C56:6	0.0123	n.s.	n.s.
TAG C60:5	0.0004	n.s.	n.s.
TAG C58:5	0.0001	n.s.	n.s.
TAG C58:4	0.0002	n.s.	n.s.
TAG C60:3	0.0024	n.s.	n.s.
PC aa C36:4	0.0452	n.s.	n.s.
PC aa C36:5	0.0142	n.s.	n.s.
PC aa C30:0	0.0768	n.s.	n.s.
PC aa C26:0	n.s.	n.s.	n.s.
PC aa C36:3	0.0344	n.s.	n.s.
PC ae C44:3	0.0000	n.s.	n.s.
PC ae C42:5	0.0000	n.s.	n.s.
PC ae C44:6	0.0000	n.s.	n.s.
PC ae C30:1	0.0039	n.s.	n.s.
PC ae C30:2	0.0175	n.s.	n.s.
Lyso PC C06:0	0.0069	n.s.	0.0881
Lyso PC C10:0	0.0000	n.s.	0.0870
Lyso PC 16:1	0.0000	n.s.	n.s.
Lyso PC C18:0	0.0000	n.s.	n.s.
Lyso PC C20:4	0.0000	n.s.	n.s.
SM C22:3	0.0139	n.s.	n.s.
SM C16:0	n.s.	n.s.	n.s.
SM C20:2	n.s.	n.s.	n.s.
SM C18:0	n.s.	n.s.	n.s.
SM C22:1 OH	n.s.	n.s.	n.s.
C08	n.s.	n.s.	n.s.
C16	0.0002	n.s.	0.0715
C16:1	0.0001	n.s.	0.0713
C16:2	0.0000	n.s.	0.0464
C18:1 OH	0.0000	n.s.	n.s.

Supplemental Figure S8: Influence of acitretin treatment in the brain of wild type mice regarding lipid species with maximal fold change in 5xFAD mice. Fold changes of single triacylglyceride (A), phosphatidylcholine (B), phosphatidylcholine-plasmalogens (C), lyso-phosphatidylcholine (D), sphingomyeline (E) and carnitine (F) species in brains of 5xFAD mice after acitretin treatment in comparison to controls are shown as volcano plots at the top. Structure and labeling of the volcano blots are according to figure 2. Changes in the lipid species with the greatest fold change in 5xFAD brain samples after acitretin treatment are shown as bar chart at the bottom (black bars). These top five changes lipid species were analyzed in control- or acitretin treated wild type mice in an initial experiment and the resulting fold changes are presented as grey bars in the bar chart at the bottom for each lipid class, respectively. (G) Two-way ANOVA of lipid species presented in A-F. Three null hypotheses were tested at the same time: (1) differences between the genotype, (2) differences between control and acitretin treatment, (3) does the acitretin treatment effect depends on the genotype variable (interaction effect) (n.s.: $p \geq 0.1$).

Table S1: Q1/Q3 masses, declustering potentials (DP), and collision energy (CE) for the analyzed metabolites.

metabolite	Q1 mass (Da)	Q3 mass (Da)	DP (volts)	CE (volts)
**C0**	162.1	85.1	61	27
**C2**	204.1	85.1	41	27
**C3**	218.1	85.1	46	29
**C03 OH**	234.1	85.1	53	30
**C3:1**	216.1	85.1	49	27
**C4**	232.2	85.1	46	29
**C4 OH**	248.1	85.1	55	32
**C4:1**	230.1	85.1	52	29
**C5**	246.2	85.1	46	29
**C5 M DC**	290.2	85.1	63	37
**C5 OH**	262.2	85.1	58	33
**C5:1**	244.2	85.1	55	31
**C5:1 DC**	274.1	85.1	60	35
**C6**	260.2	85.1	56	27
**C6 OH**	276.2	85.1	61	35
**C6:1**	258.2	85.1	57	33
**C7 DC**	304.2	85.1	66	39
**C8**	288.2	85.1	66	33
**C8:1**	286.2	85.1	63	37
**C9**	302.2	85.1	66	39
**C10**	316.2	85.1	56	37
**C10:1**	314.2	85.1	68	40
**C10:2**	312.2	85.1	67	40
**C12**	344.3	85.1	73	44
**C12 DC**	374.3	85.1	86	45
**C12:1**	342.3	85.1	73	44
**C14**	372.3	85.1	86	45
**C14:1**	370.3	85.1	78	47
**C14:1 OH**	386.3	85.1	81	50
**C14:2**	368.3	85.1	78	47
**C14:2 OH**	384.3	85.1	81	49
**C16**	400.3	85.1	84	51
**C16 OH**	416.3	85.1	87	53
**C16:1**	398.3	85.1	84	51
**C16:1 OH**	414.3	85.1	87	53
**C16:2**	396.3	85.1	83	51
**C16:2 OH**	412.3	85.1	86	53
**C18:0**	428.4	85.1	96	63
**C18:1**	426.4	85.1	89	55
**C18:1 OH**	442.4	85.1	92	57
**C18:2**	424.3	85.1	89	54

**Carnitine Std 1**	291.2	85.1	66	33
**Carnitine Std 2**	403.3	85.1	84	51
**Lyso PC 06:0**	356.2	184	72	31
**Lyso PC 10:0**	412.3	184	76	30
**Lyso PC 12:0**	440.4	184	78	30
**Lyso PC 14:0**	468.3	184	79	30
**Lyso PC 16:0**	496.3	184	81	31
**Lyso PC 16:1**	494.3	184	79	30
**Lyso PC 17:0**	510.3	184	82	31
**Lyso PC 18:0**	524.3	184	85	31
**Lyso PC 18:1**	522.3	184	82	31
**Lyso PC 18:2**	520.3	184	82	31
**Lyso PC 18:3**	518.4	184	82	31
**Lyso PC 20:0**	552.4	184	89	31
**Lyso PC 20:3**	546.3	184	87	31
**Lyso PC 20:4**	544.3	184	85	31
**Lyso PC 20:5**	542.4	184	84	31
**Lyso PC 22:0**	580.5	184	93	32
**Lyso PC 22:6**	568.4	184	90	32
**Lyso PC 24:0**	608.4	184	96	33
**Lyso PC 26:0**	636.5	184	101	34
**Lyso PC 26:1**	634.4	184	98	33
**Lyso PC 28:0**	664.5	184	105	35
**Lyso PC 28:1**	662.5	184	103	35
**Lyso PC Std**	538.6	184	96	40
**PC aa C20:0**	566.2	184	94	31
**PC aa C24:0**	622.4	184	98	33
**PC aa C26:0**	650.5	184	103	35
**PC aa C28:0**	678.5	184	106	35
**PC aa C28:1**	676.5	184	106	35
**PC aa C30:0**	706.5	184	114	37
**PC aa C30:2**	702.5	184	111	37
**PC aa C32:0**	734.6	184	119	39
**PC aa C32:1**	732.6	184	118	39
**PC aa C32:2**	730.5	184	117	38
**PC aa C32:3**	728.5	184	117	38
**PC aa C34:1**	760.6	184	125	41
**PC aa C34:2**	758.6	184	123	40
**PC aa C34:3**	756.6	184	123	40
**PC aa C34:4**	754.5	184	122	40
**PC aa C36:0**	790.6	184	131	43
**PC aa C36:1**	788.6	184	131	43
**PC aa C36:2**	786.6	184	130	42
**PC aa C36:3**	784.6	184	130	42

**PC aa C36:4**	782.6	184	129	42
**PC aa C36:5**	780.6	184	128	42
**PC aa C36:6**	778.5	184	128	42
**PC aa C38:0**	818.7	184	138	45
**PC aa C38:1**	816.7	184	138	45
**PC aa C38:3**	812.6	184	136	44
**PC aa C38:4**	810.6	184	136	44
**PC aa C38:5**	808.6	184	136	44
**PC aa C38:6**	806.6	184	135	44
**PC aa C40:0**	846.7	184	145	47
**PC aa C40:1**	844.7	184	145	47
**PC aa C40:2**	842.7	184	144	47
**PC aa C40:3**	840.7	184	144	47
**PC aa C40:4**	838.6	184	144	47
**PC aa C40:5**	836.6	184	143	47
**PC aa C40:6**	834.6	184	143	46
**PC aa C42:0**	874.7	184	154	50
**PC aa C42:1**	872.7	184	153	50
**PC aa C42:2**	870.7	184	153	50
**PC aa C42:4**	866.7	184	152	50
**PC aa C42:5**	864.7	184	151	49
**PC aa C42:6**	862.6	184	150	49
**PC aa C44:0**	902.7	184	163	50
**PC aa C48:0**	958.8	184	170	50
**PC aa Std**	454.4	184	76	39
**PC ae C30:0**	692.6	184	111	37
**PC ae C30:1**	690.5	184	109	36
**PC ae C30:2**	688.5	184	108	36
**PC ae C32:1**	718.6	184	115	38
**PC ae C32:2**	716.6	184	114	37
**PC ae C34:0**	748.6	184	122	40
**PC ae C34:1**	746.6	184	121	39
**PC ae C34:2**	744.6	184	120	39
**PC ae C34:3**	742.6	184	120	39
**PC ae C36:0**	776.7	184	128	42
**PC ae C36:1**	774.6	184	127	41
**PC ae C36:2**	772.6	184	127	41
**PC ae C36:3**	770.6	184	126	41
**PC ae C36:4**	768.6	184	126	41
**PC ae C36:5**	766.6	184	125	41
**PC ae C38:0**	804.7	184	135	44
**PC ae C38:1**	802.7	184	134	44
**PC ae C38:2**	800.7	184	133	43
**PC ae C38:3**	798.6	184	133	43

**PC ae C38:4**	796.6	184	133	43
**PC ae C38:5**	794.6	184	132	43
**PC ae C38:6**	792.6	184	132	43
**PC ae C40:0**	832.7	184	142	46
**PC ae C40:1**	830.7	184	141	46
**PC ae C40:2**	828.7	184	141	46
**PC ae C40:3**	826.7	184	140	46
**PC ae C40:4**	824.7	184	140	45
**PC ae C40:5**	822.6	184	139	45
**PC ae C40:6**	820.6	184	139	45
**PC ae C42:0**	860.8	184	150	49
**PC ae C42:1**	858.7	184	141	46
**PC ae C42:2**	856.7	184	149	48
**PC ae C42:3**	854.7	184	148	48
**PC ae C42:4**	852.7	184	148	48
**PC ae C42:5**	850.7	184	147	48
**PC ae C44:3**	882.7	184	156	51
**PC ae C44:4**	880.7	184	156	51
**PC ae C44:5**	878.7	184	155	51
**PC ae C44:6**	876.7	184	154	50
**PC ee Std**	594.6	184	74	57
**SM 18:1/14:1 OH**	689.6	184	108	36
**SM 18:1/16:0**	703.6	184	112	37
**SM 18:1/16:1**	701.6	184	111	37
**SM 18:1/16:1 OH**	717.6	184	114	38
**SM 18:1/18:0**	731.6	184	117	38
**SM 18:1/18:1**	729.6	184	117	38
**SM 18:1/20:2**	755.6	184	123	40
**SM 18:1/22:1 OH**	801.7	184	134	43
**SM 18:1/22:2 OH**	799.7	184	133	43
**SM 18:1/22:3**	781.6	184	129	42
**SM 18:1/24:0**	815.7	184	137	45
**SM 18:1/24:1**	813.7	184	137	45
**SM 18:1/24:1 OH**	829.7	184	141	46
**SM 18:1/26:0**	843.7	184	145	47
**SM 18:1/26:1**	841.7	184	144	47
**SM Std**	563.6	184	76	31
**C48:0**	824.8	551.8	100	25
**C50:3**	846.8	573.8	100	25
**C50:2**	848.8	575.8	100	25
**C50:1**	850.8	577.8	100	25
**C50:0**	852.8	579.8	100	25
**C52:6**	868.7	595.7	100	25
**C52:5**	870.8	597.8	100	25

**C52:4**	872.8	599.8	100	25
**C52:3**	874.8	601.8	100	25
**C52:2**	876.8	603.8	100	25
**C52:1**	878.8	605.8	100	25
**C52:0**	880.8	607.8	100	25
**C54:9**	890.7	595.7	100	25
**C54:8**	892.7	597.7	100	25
**C54:7**	894.8	599.8	100	25
**C54:6**	896.8	601.8	100	25
**C54:5**	898.8	625.8	100	25
**C54:4**	900.8	627.8	100	25
**C54:3**	902.8	629.8	100	25
**C54:2**	904.8	631.8	100	25
**C54:1**	906.9	633.9	100	25
**C56:8**	920.8	625.8	100	25
**C56:7**	922.8	627.8	100	25
**C56:6**	924.8	629.8	100	25
**C56:5**	926.8	631.8	100	25
**C56:4**	928.8	633.8	100	25
**C56:3**	930.9	635.9	100	25
**C56:2**	932.9	659.9	100	25
**C56:1**	934.9	661.9	100	25
**C58:7**	950.8	655.8	100	25
**C58:6**	952.8	657.8	100	25
**C58:5**	954.8	659.8	100	25
**C58:4**	956.9	661.9	100	25
**C58:3**	958.9	663.9	100	25
**C58:2**	960.9	659.9	100	25
**C60:6**	980.9	685.9	100	25
**C60:5**	982.9	687.9	100	25
**C60:4**	984.9	689.9	100	25
**C60:3**	986.9	687.9	100	25
**TAG Std**	829.4	523.3	100	25
